# An increase in ER stress and unfolded protein response in iPSCs-derived neuronal cells from neuronopathic Gaucher disease patients

**DOI:** 10.1038/s41598-024-59834-6

**Published:** 2024-04-22

**Authors:** Tanapat Pornsukjantra, Nongluk Saikachain, Nareerat Sutjarit, Arthaporn Khongkrapan, Alisa Tubsuwan, Kanit Bhukhai, Thipwimol Tim-Aroon, Usanarat Anurathapan, Suradej Hongeng, Nithi Asavapanumas

**Affiliations:** 1 Program in Translational Medicine, Faculty of Medicine Ramathibodi Hospital, Mahidol University, Bangkok, 10400 Thailand; 2 Chakri Naruebodindra Medical Institute, Faculty of Medicine Ramathibodi Hospital, Mahidol University, Bang Pla, Bang Phli, Samut Prakan 10540 Thailand; 3 Graduate Program in Nutrition, Faculty of Medicine Ramathibodi Hospital, Mahidol University, Bangkok, 10400 Thailand; 4 Division of Medical Genetics, Department of Pediatrics, Faculty of Medicine Ramathibodi Hospital, Mahidol University, Bangkok, 10400 Thailand; 5 Institute of Molecular Biosciences, Mahidol University, Nakhon Pathom, 73170 Thailand; 6 Department of Physiology, Faculty of Science, Mahidol University, Bangkok, 10400 Thailand; 7 Department of Pediatrics, Faculty of Medicine Ramathibodi Hospital, Mahidol University, Bangkok, 10400 Thailand

**Keywords:** Gaucher disease, iPSCs-derived neurons, ER stress, UPR, LSDs, Biological techniques, Genetics, Molecular biology, Neuroscience, Physiology, Stem cells, Diseases, Molecular medicine

## Abstract

Gaucher disease (GD) is a lysosomal storage disorder caused by a mutation in the *GBA1* gene, responsible for encoding the enzyme Glucocerebrosidase (GCase). Although neuronal death and neuroinflammation have been observed in the brains of individuals with neuronopathic Gaucher disease (nGD), the exact mechanism underlying neurodegeneration in nGD remains unclear. In this study, we used two induced pluripotent stem cells (iPSCs)-derived neuronal cell lines acquired from two type-3 GD patients (GD3-1 and GD3-2) to investigate the mechanisms underlying nGD by biochemical analyses. These iPSCs-derived neuronal cells from GD3-1 and GD3-2 exhibit an impairment in endoplasmic reticulum (ER) calcium homeostasis and an increase in unfolded protein response markers (BiP and CHOP), indicating the presence of ER stress in nGD. A significant increase in the BAX/BCL-2 ratio and an increase in Annexin V-positive cells demonstrate a notable increase in apoptotic cell death in GD iPSCs-derived neurons, suggesting downstream signaling after an increase in the unfolded protein response. Our study involves the establishment of iPSCs-derived neuronal models for GD and proposes a possible mechanism underlying nGD. This mechanism involves the activation of ER stress and the unfolded protein response, ultimately leading to apoptotic cell death in neurons.

## Introduction

Gaucher disease (GD) is one of the most common lysosomal storage diseases (LSDs) worldwide^1^. The symptoms of GD are heterogeneous, varying from patient to patient. These can include hepatosplenomegaly, bone manifestations, pancytopenia, and neurological defects^2^. Clinically, GD is categorized into three subtypes, based on the presence and severity of neurological symptoms. Type-1 GD, which is non-neuronopathic, often manifests during adolescence or early adulthood and does not involve neurological symptoms. In contrast, both type-2 and type-3 GD present with neurological symptoms, also called neuronopathic Gaucher disease (nGD)^2,3^. It is generally known that GD patients harbor autosomal recessive mutations on the *GBA1* gene, encoded for acid-beta-glucosidase or glucocerebrosidase (GCase) enzyme^4,5^. This enzyme is crucial for the degradation of lysosomal substrates, specifically glucosylceramide (GlcCer). The mutations can lead to the production of defective GCase with diminished enzymatic activity^2^. Among more than 700 reported *GBA1* pathogenic variants, the most common variant which associated with the nGD is a L444P mutation^6^. Patients carrying L444P mutation exhibit a heterogenous phenotype^2^, including astrogliosis and neuronal loss^7,8^. However, it is still unclear what the underlying mechanism of the pathological features observed in the brains of GD patients is, as well as why there is heterogeneity among GD types^5,9^. A deeper understanding of the pathophysiology of GD will be invaluable in determining the factors contributing to the variability of its symptoms.

Endoplasmic reticulum (ER) stress and the dysfunctional unfolded protein response (UPR) are widely recognized as significant contributors to neurological symptoms in various diseases^10,11^. They have been linked to neurodegenerative conditions, including Parkinson’s disease, Alzheimer’s disease, and other LSDs^12–14^. The disruption of ER homeostasis and function can result from abnormal Ca^2+^ regulation, protein dysregulation, or an accumulation of unfolded/misfolded proteins, leading to ER stress. When unfolded or misfolded proteins accumulate, a set of signal transduction pathways known as UPR is activated. The UPR is initiated by three ER transmembrane sensors: inositol-requiring transmembrane kinase/endoribonuclease 1 (IRE1), activating transcription factor 6 (ATF6), and double-stranded RNA-dependent protein kinase-like eukaryotic initiation factor 2α kinase (PERK), triggered by the dissociation of the binding immunoglobulin protein (BiP)^15^. These UPR pathways initially attempt to restore ER homeostasis, promoting cell survival. If ER stress becomes overwhelming and homeostasis cannot be restored, UPR signaling may instead trigger apoptosis^16^. C/EBP homology protein (CHOP), a transcription factor, plays a key role in the UPR-related apoptosis pathway. Increased CHOP levels lead to the upregulation of pro-apoptotic protein BAX, ultimately resulting in apoptosis^17^.

In GD, variants in the *GBA1* gene lead to the retention of misfolded proteins in the ER. This retention triggers BiP for refolding, and the refolded proteins are subsequently transported to the lysosome^18^. Studies have demonstrated that the retention of misfolded GCase triggers ER stress and dysregulated UPR in fibroblasts of GD type patients and in the brain of mouse models of GD^19–22^. However, the evidence for ER stress and dysregulated UPR triggering apoptosis in nGD remains inconclusive, particularly given the absence of increased ER stress and UPR in primary mouse neurons and astrocyte culture models of nGD^23^.

In this study, we investigated the role of ER stress and dysregulated UPR-mediated apoptosis in nGD using induced pluripotent stem cell (iPSCs)-derived cortical neurons. Our generated iPSCs lines from two type-3 nGD patients who exhibited early signs of neurological involvement^24,25^ were used in this study. These iPSCs-derived neuronal cells from both GD patients displayed disease phenotypes, including significantly reduced GCase activities and protein levels. Furthermore, we observed an increase in ER stress and dysregulated UPR in iPSCs-derived neuronal cells from nGD patients. This UPR activation led to apoptosis, resulting in an increased number of apoptotic cells in iPSCs-derived neuronal cells from nGD patients.

## Methods

### Study approval

All experiments conducted in this study followed the guidelines and regulations approved by the Ethics Committee on Human Rights Related to Research Involving Human Subjects at the Faculty of Medicine Ramathibodi Hospital, Mahidol University, Thailand (Approval number: MURA2019/447 and MURA2023/79).

### Cell culture

The iPSCs were cultured according to a previously described protocol using our in-house Essential 8 medium^25^. iPSCs from passages 25–35 were utilized for neural induction. The neural induction medium consisted of an equal ratio of DMEM F/12 and advanced neurobasal medium (Gibco, USA), 1X GlutaMAX (Gibco, USA), 1X B27 supplement without vitamin A (Gibco, USA), 1X N2 supplement (Gibco, USA), 10 µM SB431542 (Sigma-Aldrich, USA), 0.1 µM LDN193189, and 0.1X penicillin/streptomycin for differentiating iPSCs into neural progenitor cells (NPCs). Afterwards, neural expansion medium was used to culture the NPCs further (1:1 ratio of DMEM F/12 and Advanced neurobasal medium, 1X GlutaMAX, 1X B27 supplement without vitamin A, 1X N2 supplement, 20 ng/µl FGF-2, 20 ng/µl EGF, and 0.1X penicillin/streptomycin). The terminal differentiation was achieved on NPC passage 4–5 using neural differentiation medium, consisting of 1:1 ratio of DMEM F/12 and Advanced neurobasal medium, 1X GlutaMAX, 1X B27 supplement without vitamin A, 1X N2 supplement, 50 µM DB-cAMP, 200 µM Ascorbic acid, 20 ng/ml BDNF (Peprotech, USA), and 10 ng/ml GDNF (Peprotech, USA)**.**

### Fluorescent staining

Cells were plated on a 96-well black plate pre-coated with poly-L-ornithine/laminin at 15,000 cells/cm^2^ and maintained for 7 days. The cells were cleaned three times with PBS after being fixed with 4% paraformaldehyde for 20 min. Following permeabilization with 0.1% Triton X 100 in PBS for 15 min at 37 °C, the cells were then blocked for an hour with the blocking solution of 2% bovine serum albumin (Sigma Aldrich, USA) in PBS. Cells were incubated overnight at 4 °C with antibodies against human Nanog (1:200, Santa Cruz Biotechnology (SC33759)), Tra-1-60 (1:200, Santa Cruz Biotechnology (SC21705)), PAX6 (1:400, Invitrogen (MA1-109)), Tuj1 (1:400, Santa Cruz Biotechnology (SC51670)), S100B (1:100, Abcam (ab52642)), or Synaptophysin (1:200, Abcam (ab32127)) followed by incubating with the appropriate fluorescent secondary antibody (1:1000, Alexa Fluor 488 and 555, Cell Signaling Technology (4409S, 4413S and A11001) for 1 h at room temperature. Hoechst 33342 (Invitrogen (H3570)) in PBS at a concentration of 1:2,500 was used to stain the cell nuclei for five minutes at room temperature. Cells were examined with Zeiss LSM 900 confocal microscope (Carl Zeiss, Germany). For the analysis of fluorescent intensity, the mean intensity over imaging field were measured using ImageJ (ImageJ, USA). After background subtraction, fluorescent intensity was normalized to the number of cells in the field. Data are presented as the normalized intensity compared to the control group. For the lysosome and acidic organelles labeling experiment, cells were incubated with either 1 µM SiR-lysosome kit (Spirochrome), a fluorophore that specifically binds to capthesin D, or 1 µg/ml of acridine orange combined with Hoechst 33,342 (1:500, Invitrogen (H3570)) in HBSS buffer for 15–30 min at 37 °C. The living-stained organelles were examined with Opera Phenix high-content screening system (PerkinElmer, USA) at 37 °C with 5% CO_2_. The analysis of fluorescent intensity was performed using Harmony High-Content Imaging and Analysis software (PerkinElmer, USA). Regions of interests (ROIs) surrounding the soma were automatically drawn by Harmony software with background ROIs drawn in the empty area surrounding the soma. After background subtraction, the mean fluorescent intensity was normalized to the number of nuclei and data are presented as the normalized intensity comparted to the control group.

### Intracellular calcium measurement

Cells were plated on a 96-well black plate pre-coated with poly-L-ornithine/laminin at a density of 15,000 cells/cm^2^ and maintained for 7 days. Subsequently, the cells were incubated with a calcium indicator dye (Fluo-8 Calcium Flux Assay Kit, ab112129, Abcam, Cambridge, UK) for 1 h. After the incubation with the dye, the cells were kept in Hanks’ Balanced Salt Solution (HBSS) buffer. To assess the increase in intracellular calcium induced by caffeine, 10 mM of caffeine (Sigma-Aldrich, USA) in HBSS was added to the cells. In some experiments, 40 µM of cyclopiazonic acid (Sigma-Aldrich, USA) in HBSS was pre-incubated to deplete calcium in the endoplasmic reticulum . Intracellular calcium signaling was recorded at a frame rate of 2 Hz using a Zeiss LSM 900 confocal microscope (Carl Zeiss, Germany).

### Western blot analysis

Total protein was extracted from the NPCs and neurons using RIPA lysis buffer (Thermo Fisher Scientific, USA) with protease inhibitors. Protein concentrations were measured using the BCA assay (Thermo Fisher Scientific, USA) following the manufacturer’s instructions. Subsequently, 50 µg of proteins were separated by electrophoresis on SDS-PAGE and transferred onto a nitrocellulose membrane. After transfer, nitrocellulose membranes were cut to the corresponding size of the target protein for incubating with primary antibodies, without the need for stripping and reprobing procedures. Images of all replicates are provided in the Supplementary information file. Primary antibodies were incubated overnight at 4 °C at a concentration of 1:1,000 (GBA1 (Abcam (Ab55080)), LAMP1 (Abcam (Ab25630)), LC3 (Cell Signaling Technology (12,741), BiP (Cell Signaling Technology (3177)), CHOP (Cell Signaling Technology (2895)), BAX (Cell Signaling Technology (2772)), BCL-2 (Cell Signaling Technology (3498)). Following this, a secondary antibody conjugated to horseradish peroxidase at a concentration of 1:10,000 was incubated for 1 h at room temperature. The membranes were then exposed in the Bio-Rad ChemiDoc system, and the analyses were performed using Image Lab software.

### Real time PCR

Isolation of total RNA from NPCs and neurons was achieved using Total RNA isolation kit (Geneaid, Taiwan) with DNAse treatment according to the manufacturer’s protocol. iScript cDNA synthesis kit (Biorad, USA) was used to perform the cDNA synthesis following the manufacturer’s protocol for 1 µg of RNA. Each reaction of iScript qPCR Master Mix (Biorad, USA) was supplied with 2 µl of cDNAs at 1:20 dilution ratio, and 5 µM of each primer. The qPCRs were performed according to the manufacturer’s instructions using Biorad CFX96 Touch Real-Time PCR Detection System. The ΔCt method was used to calculate the relative mRNA expression; GAPDH was used as a reference gene. Total RNA isolation from NPCs and neurons was achieved using the Total RNA Isolation Kit (Geneaid, Taiwan) with DNAse treatment following the manufacturer’s protocol. For cDNA synthesis, the iScript cDNA Synthesis Kit (Bio-Rad, USA) was employed, following the manufacturer’s protocol, using 1 µg of RNA as the starting material. Each reaction of iScript qPCR Master Mix (Bio-Rad, USA) included 2 µl of cDNAs at a 1:20 dilution ratio and 5 µM of each primer. The qPCRs were performed according to the manufacturer’s instructions using the Bio-Rad CFX96 Touch Real-Time PCR Detection System. The ΔCt method was employed to calculate the relative mRNA expression, with GAPDH serving as the reference gene. The primers used for the experiments are specified in Supplementary Table 1.

### GCase activity assay

A GCase activity assay was performed using the degradation of 4-methylumbelliferyl β-D-glucopyranoside. Cells from the culture were lysed using an ultrasonic homogenizer (Omni-Ruptor 4000). The protein concentration of the lysates was determined using the BCA assay (Thermo Fisher Scientific, USA). A total of 30 µg of lysate proteins were incubated in phosphate-citrate (McIlvaine’s) buffer, pH 5.4, with 4-methylumbelliferyl β-D-glucopyranoside substrates at 37 °C for 30 min. The fluorescent signal was measured using a plate reader, and the enzyme activity was calculated.

### Whole exome sequencing analysis

Genomic DNA was extracted from peripheral blood samples of the patients using the Gentra^®^ Puregene^®^ kit (QIAGEN^®^, Germany). For research of variants in the modifier genes, Whole Exome Sequencing (WES) was performed using the Illumina HiSeq4000 sequencer by Macrogen^®^ (Seoul, Republic of Korea), then the WES data was quality checked and analyzed. Agilent’s SureSelect (V5 + UTR) was used for target enrichment, and DNA sequencing was conducted in 100-bp Pair End mode, achieving a target region coverage of 125x. The obtained data underwent quality control using the FastQC package. Read alignment against the hg19 reference genome from the UCSC genome browser database was performed using the Burrows-Wheeler Aligner (BWA version 0.5.9). Databases used for determining possible pathogenicity and MAF of the allele identified were as follow: ClinVar (https://www.ncbi.nlm.nih.gov/clinvar); the Human Genome Mutation Database (http://www.biobase-international.com); population database including the gnomAD, dbSNP version 142, 1000Genome phase3, the Exome Variant Server (version ESP6500SI V2; https://evs.gs.washington.edu/EVS/), and the T-REx database (Thai genome database of 2000 WES, https://trex.nbt.or.th. Analysis of WES data utilized VCF files and the online software BaseSpace Variant Interpreter program (https://variantinterpreter.informatics.illumina.com). For the analysis, gene lists associated with ER stress and genes known to be linked with Gaucher disease from Dombroski et al. and Davidson et al. were employed to filter for the variants^15,16^.

### Flow cytometry

iPSCs and neural progenitor cells collected from the culture were washed twice with cold PBS and then fixed for 15 min at room temperature in 4% paraformaldehyde. Subsequently, the cells were permeabilized for 10 min with 0.1% Triton-X 100 in PBS. Annexin V and propidium iodide antibodies were incubated for 1 h at 4 °C, with three rounds of washing in cold PBS in between. Flow cytometry analysis was performed using BD Accuri C6 and BD FACSuite software. The total cell count was obtained by harvesting the culture medium and dissociating the neurons for 10 min at 37 °C using Accutase dissociation solution (Sigma-Aldrich, USA). The FITC-Annexin V apoptosis detection kit (BD Bioscience, USA) was used following the manufacturer’s protocol. Flow cytometry analysis was conducted using BD Accuri C6 and BD FACSuite software. Unstained and single-stained cells were used as controls to gate the analytical quadrants.

### Statistical analysis

Data analysis was conducted using GraphPad Prism 10 software (GraphPad Software, Inc) and MATLAB. Sample size estimation was based on previous studies involving iPSCs-derived neuronal cells for neurodegenerative research ^26,27^ considering a power of 0.8 and a type I error rate of 0.05. The data were presented as median ± interquartile range. The normality of data distribution in each dataset was assessed using the one-sample Kolmogorov–Smirnov test. Two-sample Kolmogorov–Smirnov test was used for comparing two data distributions. To evaluate statistical significance among three independent groups, we employed the Kruskal–Wallis test followed by Uncorrected Dunn’s test was used for comparing three groups.

### Ethics approval

Human ethical approval for sample collection, methods, and experiments was obtained from the Ethics Committee on Human Rights Related to Research Involving Human Subjects, Faculty of Medicine Ramathibodi Hospital, Mahidol University, Thailand (Approval number: MURA2019/447 and MURA2023/79). Informed consent was obtained from all the participants.

## Results

### Properties analysis of iPSCs and NPCs lines from neuronopathic GD patients and healthy control

**Figure 1:**
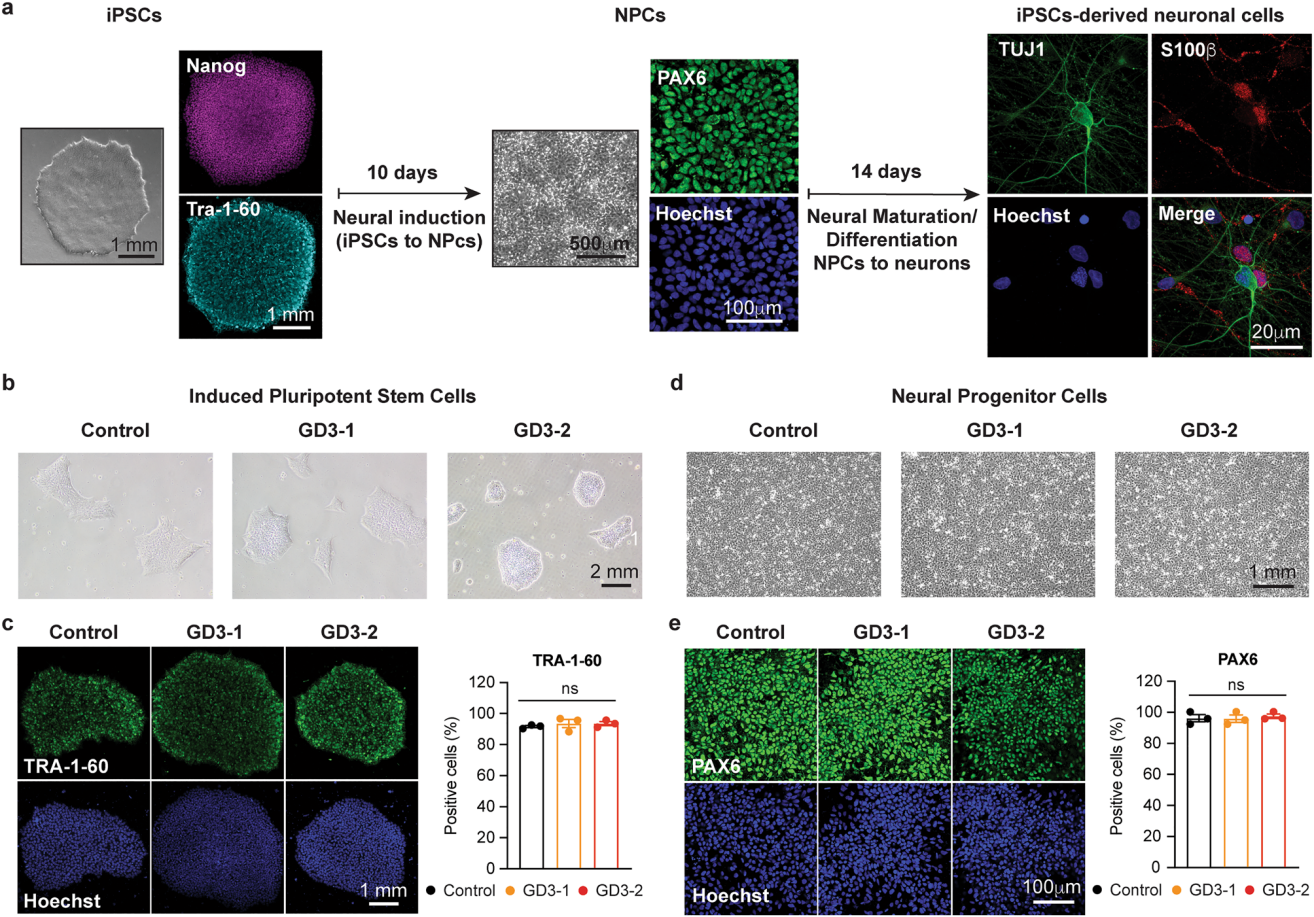
Normal iPSCs and NPCs properties in cells generated by 2 Gaucher disease patients. (**a**) Schematic representation of a three-steps neuronal differentiation protocol starting from iPSCs (left), NPCs (middle) and iPSCs-derived neuronal cells (Right) with corresponding protein marker expression. (**b**) Bright field images of iPSCs colonies in Control (left), GD3-1 (middle) and GD3-2 (right) samples. (**c**) (Left) TRA-1–60 and Hoechst fluorescence in control, GD3-1 and GD3-2 iPSCs groups. (Right) Summary of TRA-1–60 positive cells detected by flow cytometry in control, GD3-1 and GD3-2 iPSCs groups (Kruskal–Wallis test: H(2) = 1.07, *p* = 0.66, n = 3 technical replicates). (**d**) Bright field images of NPCs in Control (left), GD3-1 (middle) and GD3-2 (right) samples. (**e**) (Left) PAX6 and Hoechst fluorescence in control, GD3-1 and GD3-2 iPSCs groups. (Right) Summary of PAX6 positive cells detected by flow cytometry in control, GD3-1 and GD3-2 iPSCs groups (Kruskal–Wallis test: H(2) = 0.84, *p* = 0.70, n = 3 technical replicates).

To investigate the underlying mechanism of neuronal death in neuronopathic Gaucher disease, we employed a 3-step differentiation protocol adapted from^28^ to develop a model of cortical neurons. The schematic representation of the differentiation process is depicted in Fig. 1a. In brief, the iPSCs were cultured in a neural induction medium for 10 days to generate neural progenitor cells (NPCs), followed by neural expansion for 7 days. Finally, terminal differentiation was achieved through 14 days of neural maturation to generate neurons. Two iPSCs lines from type-3 GD patients carrying the homozygous L444P mutation (^24,25^, GD3-1 and GD3-2, respectively) were utilized in this study, while an iPSCs line from a healthy volunteer (^29^, Control) served as the control condition.

Firstly, we compared the properties of iPSCs and NPCs among the three cell lines during differentiation to ensure that each cell line was at the same stage at the beginning of differentiation process. Figure 1b,c illustrate similar iPSCs morphology and the number of iPSCs-specific marker, TRA-1-60, positive cells among the three cell lines. After confirming similar stem cell properties, the cells were induced to NPCs. Figure 1d,e represent similar NPC morphology and the number of NPC-specific marker, PAX6, positive cells among the three cell lines. These results suggest that all of the three cell lines exhibit comparable morphologies and levels of both markers.

### Decreased GAD67 mRNA expression and synaptogenesis in iPSCs-derived neuronal cells from neuronopathic GD patients

**Figure 2:**
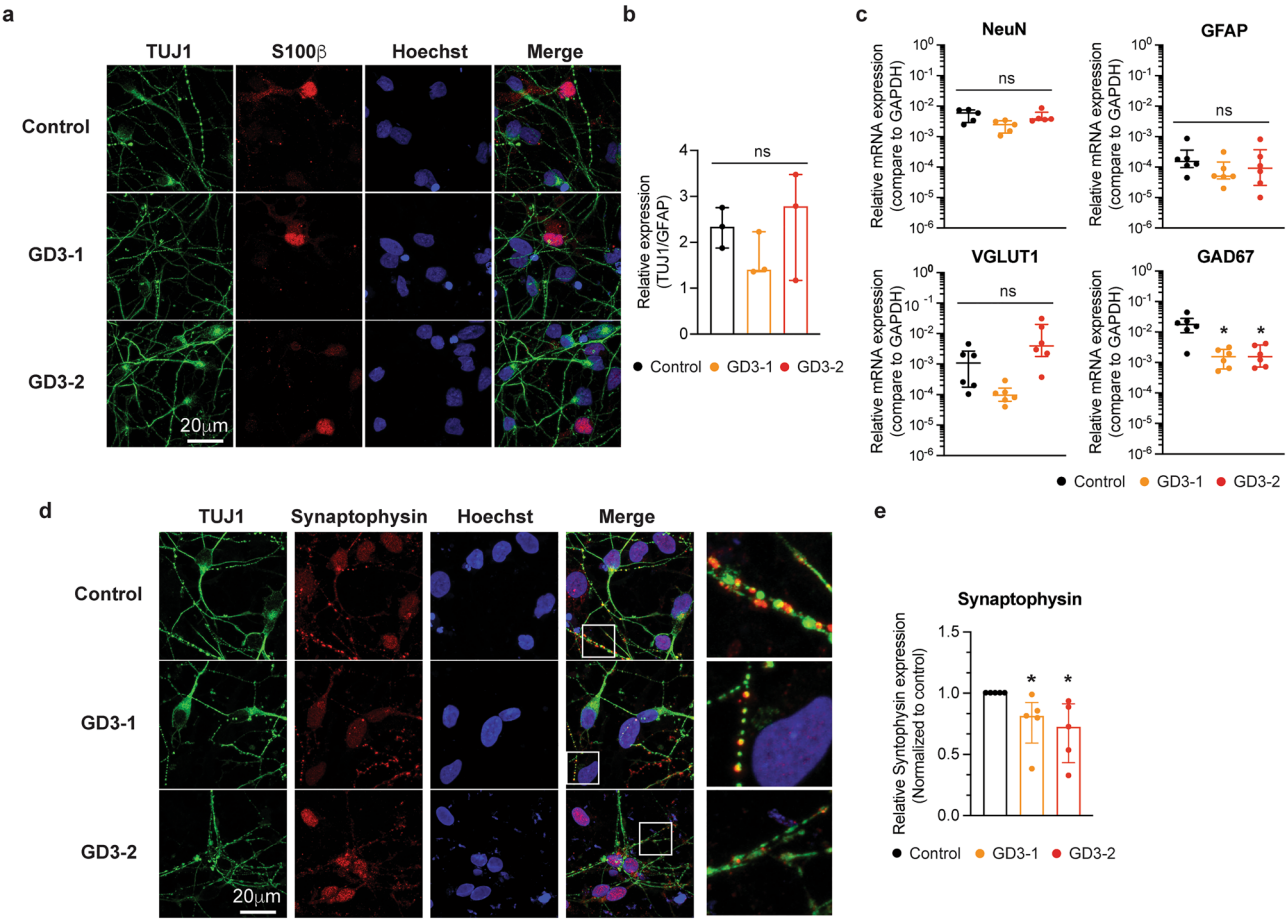
Characterization of neuronal subtype differentiation and synaptic marker in iPSCs-derived neuronal cells from GD patients. (**a**) Representative images of TUJ1, S100b, Hoechst and merge fluorescence in iPSCs-derived neuronal cells from control (top), GD3-1 (middle) and GD3-2 (bottom) samples. (**b**) Summary of the relative expression of TUJ1 over GFAP immunofluorescence per cell in GD3-1 and GD3-2 compared to control group (Kruskal–Wallis test: H(2) = 1.69, *p* = 0.51, n = 3 technical replicates). (**c**) Bar graphs representing the relative mRNA expression levels of NeuN (top left, Kruskal–Wallis test: H(2) = 6.02, *p* = 0.08 for Control vs. GD3-1, *p* > 0.99 for Control vs. GD3-2, n = 5 technical replicates), VGLUT1 (Kruskal–Wallis test: H(2) = 11.24, p = 0.09 for Control vs. GD3-1, *p* = 0.09 for Control vs. GD3-2, n = 6 technical replicates), GAD67 (bottom left, Kruskal–Wallis test: H(2) = 8.57, *p* = 0.01 for Control vs. GD3-1, *p* = 0.04 for Control vs. GD3-2, n = 6 technical replicates) and GFAP (bottom right, Kruskal–Wallis test: H(2) = 2.21, *p* = 0.35, n = 6 technical replicates) in iPSCs-derived neuronal cells from control, GD3-1 and GD3-2 samples. (**d**) Representative images of TUJ1, Synaptophysin, Hoechst and merge fluorescence in iPSCs-derived neuronal cells from control (top), GD3-1 (middle) and GD3-2 (bottom). e. Summary of the relative average synaptophysin immunofluorescence per cell in GD3-1 and GD3-2 compared to control group (Kruskal–Wallis test: H(2) = 9.85, *p* = 0.02 for Control vs. GD3-1, *p* < 0.01 for Control vs. GD3-2, n = 5 technical replicates).

After differentiation, the cells exhibited neuron-like and glial morphologies, as confirmed by the expression of neuron-specific beta-tubulin (Tuj1) and the glial-specific protein S100β. Figure 2a shows immunostaining for Tuj1 and S100β in iPSCs-derived neuronal cells from control, GD3-1 and GD3-2. We first test whether there was a difference in the ratio of neuronal (TUJ1) and glial (S100β) protein expression among 3 cell groups. All three cell groups expressed similar TUJ1 and S100β ratios (Fig. 2b). However, due to variations in proportions among the imaging fields, we measured the mRNA levels of markers for mature neurons (NeuN), glial cells (GFAP), as well as glutamatergic (VGLUT1) and GABAergic neuronal markers (GAD67) to compare the differentiation outcomes of each cell line. Figure 2c represents the mRNA expression of neuronal and glial lineage markers, including postmitotic neurons NeuN, glutamate transporter VGLUT1, the GABAergic neuronal marker GAD67, and the glial-specific marker GFAP among the three cell lines. Interestingly, iPSCs-derived neuronal cells from GD3-1 and GD3-2 exhibited a significant decrease in GAD67 mRNA expression compared to those from the control group. Meanwhile, the mRNA expression of NeuN, VGLUT1, and GFAP remained comparable among the three cell lines.

To characterize the synaptogenesis in iPSCs-derived neuronal cells from both the control and nGD groups, the expression of the pre-synaptic protein, synaptophysin, was compared among the three cell types. Figure 2c shows immunostaining for synaptophysin in iPSCs-derived neuronal cells from control, GD3-1, and GD3-2. At high magnification, the distribution of synaptophysin is evident in the proximity of Tuj1 at the neurite process (Fig. 2d, inset). A significant decrease in synaptophysin intensity within the neurite area was observed in GD3-1 and GD3-2 compared to the control group (Fig. 2e). Altogether, these results indicate a reduction in synaptic formation in iPSCs-derived neuronal cells in GD3-1 and GD3-2.

### Neuronal cells derived from iPSCs of nGD patients display neuronopathic GD phenotypes

**Figure 3:**
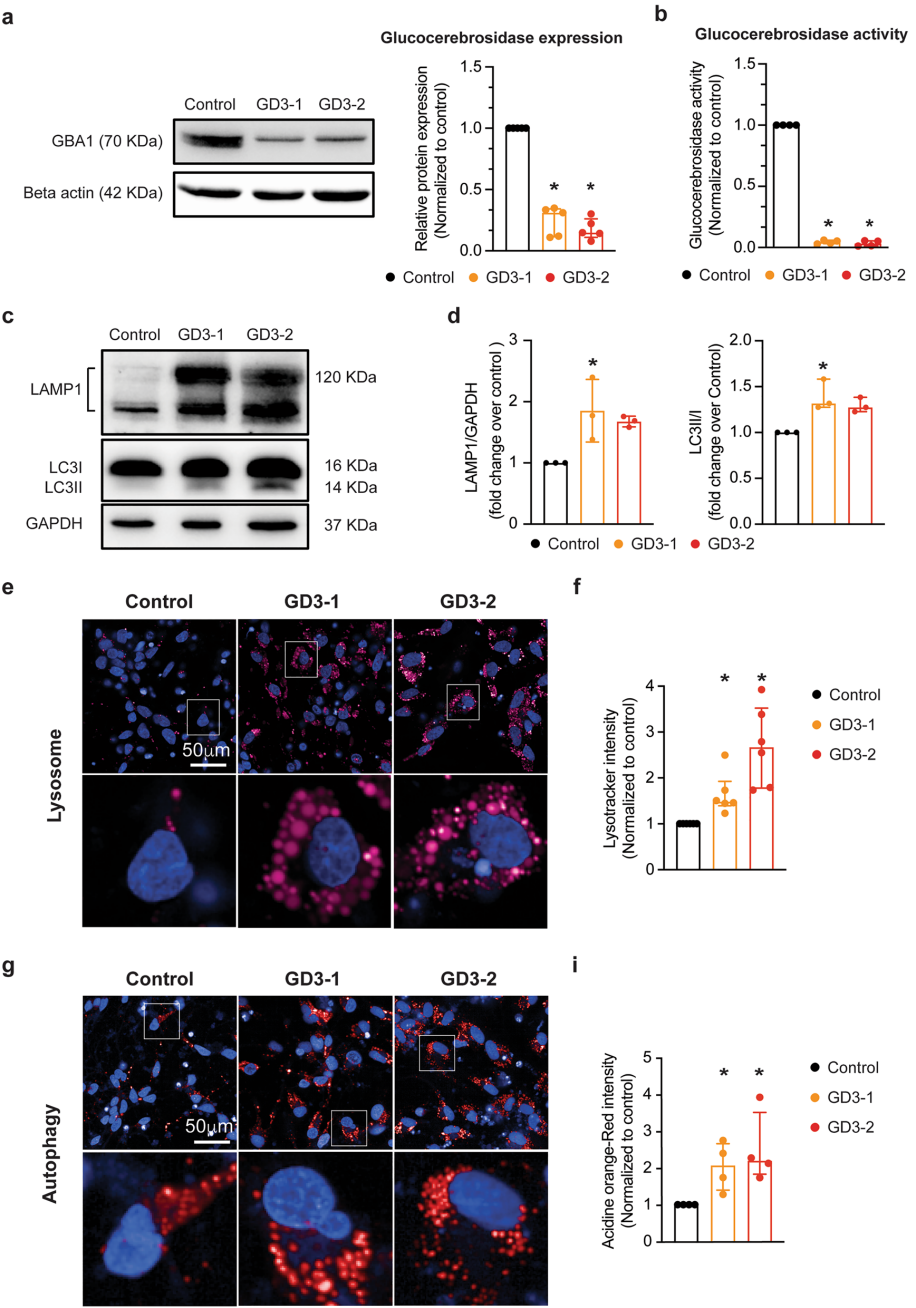
Investigation of GD phenotypes in iPSCs-derived neuronal cells acquired from neuronopathic GD. (**a**) (Left) Representative western blot images of GBA1 and beta actin from iPSCs-derived neuronal cells from control, GD3-1 and GD3-2 samples. (Right) Bar graph summarizing of the relative normalized GBA1 protein expression in GD3-1 and GD3-2 compared to control group (Kruskal–Wallis test: H(2) = 10.14, *p* = 0.04 for Control vs. GD3-1, *p* < 0.01 for Control vs. GD3-2, n = 5 technical replicates). (**b**) Bar graphs representing the relative glucocerebrosidase activity in iPSCs-derived neuronal cells (right, Kruskal–Wallis test: H(2) = 8.01, *p* = 0.04 for Control vs. GD3-1, *p* < 0.01 for Control vs. GD3-2, n = 4 technical replicates) in GD3-1 and GD3-2 compared to control group. (**c**) Representative cropped western blot images of LAMP1, LC3I/II and GAPDH from iPSCs-derived neuronal cells from control, GD3-1 and GD3-2 samples (all replicate images are included in the supplementary information file). (**d**) Bar graph summarizing of the relative normalized LAMP1(left, Kruskal–Wallis test: H(2) = 5.79, *p* = 0.02 for Control vs. GD3-1, *p* = 0.07 for Control vs. GD3-2, n = 3 technical replicates) and LC3I/II (right, Kruskal–Wallis test: H(2) = 6.16, *p* = 0.02 for Control vs. GD3-1, *p* = 0.10 for Control vs. GD3-2, n = 3 technical replicates) protein expression in GD3-1 and GD3-2 compared to control group. (**e**) (Top) Representative images of SiR lysosome staining in iPSCs-derived neuronal cells from control, GD3-1 and GD3-2 samples. (Bottom left) Expanded micrograph of boxed region demarcated in the corresponding images. (**f**) Summary of the relative average SiR lysosome staining per cell in GD3-1 and GD3-2 compared to control group (Kruskal–Wallis test: H(2) = 14.9, *p* = 0.04 for Control vs. GD3-1, *p* < 0.01 for Control vs. GD3-2, n = 6 technical replicates). (**g**) (Top) Representative images of acridine orange staining in iPSCs-derived neuronal cells from control, GD3-1 and GD3-2 samples. (Bottom) Expanded micrograph of boxed region demarcated in corresponding images. (**h**) Summary of the relative average acridine orange staining per cell in GD3-1 and GD3-2 compared to control group (Kruskal–Wallis test: H(2) = 7.69, *p* = 0.04 for Control vs. GD3-1, *p* = 0.03 for Control vs. GD3-2, n = 4 technical replicates).

To characterize the nGD phenotype, we first performed western blot analysis and enzymatic activity assays on iPSCs-derived neuronal cells to detect the levels and activity of GCase. As expected, iPSCs-derived neuronal cells exhibited a significant decrease in GCase protein levels and enzymatic activity (Fig. 3a,b). In addition, previous reports have indicated that neuron in GD exhibit autophagy defects due to lysosomal dysfunction^30^. Thus, to further validate the GD phenotype, we evaluated the amount of LAMP1 protein to assess lysosome quantity and LC3I/II protein to evaluate autophagic levels in the iPSCs-derived neuronal cells. The iPSCs-derived neuronal cells from GD3-1 showed a significant increase in both LAMP1 and LC3I/II protein levels, while cells from GD3-2 exhibited a trend towards an increase in both proteins but did not reach a significant level compared to cells from the control group (Fig. 3c,d). Additionally, we examined lysosomes staining using SiR-lysosome, which specifically binds to capthesin D, and acidic organelles using acridine orange. Interestingly, we observed a significant increase in the number of both lysosomes (Fig. 3e,f) and acidic organelles (Fig. 3g,i) in the iPSCs-derived neuronal cells from both GD groups.

### Increased Ca2+ release from intracellular stores in neuronopathic GD

**Figure 4:**
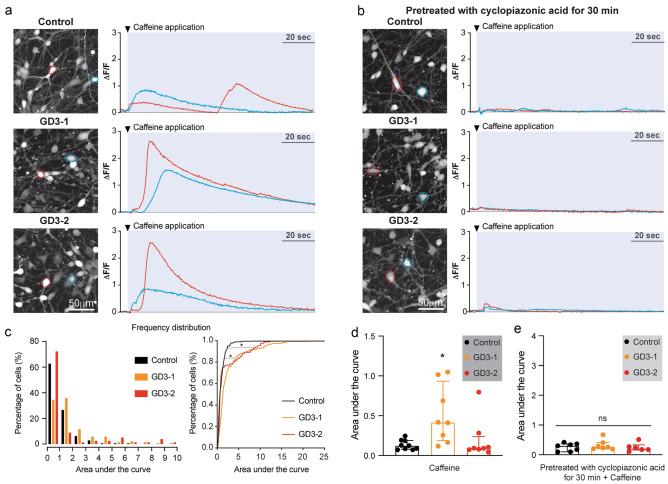
Increase in Caffeine-induced Ca^2+^ release in iPSCs derived neuronal cells from GD. (**a**) (Left) Maximum intensity projection (MIP) images of Fluor-8 fluorescence of iPSCs-derived neuronal cells from control, GD3-1 and GD3-2 samples. (Right) Two representative traces recorded from cells marked with a respective color in the MIP images corresponding to the left panel during caffeine application (indicated by tick mark). (**b**) (Left) Maximum intensity projection (MIP) image of Fluor-8 fluorescence of iPSCs-derived neuronal cells from control, GD3-1 and GD3-2 samples pretreated with cyclopiazonic acid for 30 min. (Right) Two representative traces recorded from cells marked with a respective color in the MIP images corresponding left panel during caffeine application (indicated by tick mark). (**c**) (Left) Histograms representing the distribution of area under the curve of Ca^2+^ transients during caffeine application (blue area in A) in iPSCs-derived neuronal cells from control (black), GD3-1 (orange) and GD3-2 (red) samples. (Right) Cumulative distribution of area under the curve of Ca^2+^ transients during caffeine application (blue area in A) in iPSCs-derived neuronal cells from control (black), GD3-1 (orange) and GD3-2 (red) samples (Two-sample Kolmogorov–Smirnov test: D = 0.33, *p* < 0.01 for Control vs. GD3-1; D = 0.19, *p* = 0.02 for Control vs. GD3-1, n = 177, 150 and 83 cells for control, GD3-1 and GD3-2, respectively). (**d**) Summary of the average area under the curve of Ca^2+^ transients during caffeine application per experimental in control, GD3-1 and GD3-2 groups (Kruskal–Wallis test: H(2) = 8.83, *p* = 0.02 for Control vs. GD3-1, *p* > 0.99 for Control vs. GD3-2, n = 9,8 and 8 technical replicates for Control, GD3-1 and GD3-2, respectively). e. Summary of the average area under the curve of Ca^2+^ transients during caffeine application with pretreatment with cyclopiazonic acid per experimental in control, GD3-1 and GD3-2 groups (Kruskal–Wallis test: H(2) = 0.89, *p* = 0.66, n = 7,7 and 6 technical replicates for Control, GD3-1 and GD3-2, respectively).

The involvement of ER stress in iPSCs-derived neuronal cells from nGD was initially investigated, and the ER Ca^2+^ handling was compared among healthy control, GD3-1, and GD3-2. The ryanodine receptor agonist, caffeine, was applied to measure caffeine-induced Ca^2+^ release (see Fig. 4a). To validate the specificity of caffeine-induced Ca^2+^ release, we pretreated the cells with an inhibitor of the sarcoplasmic/endoplasmic reticulum Ca^2+^-ATPase, Cyclopiazonic acid (CPA), to deplete Ca^2+^ stores in the ER. Pretreatment with CPA for 30 min abolished caffeine-induced Ca^2+^ release in iPSCs-derived neuronal cells in all cell lines (Fig. 4b). All of the recording video files are provided in the additional information section. The extent of caffeine-induced Ca^2+^ release in iPSCs-derived neuronal cells in control and nGD was estimated by calculating the area under the curve of the Ca^2+^ transient during caffeine application (blue area in Fig. 4a, left). Overall, the area under the curve of Ca^2+^ transients induced by caffeine in GD3-1 and GD3-2 significantly shifted the histogram distribution towards higher values (Fig. 4c). Figure 4D demonstrates a significant increase in caffeine-induced Ca^2+^ release in iPSCs-derived neuronal cells from nGD. Additionally, the increase in caffeine-induced Ca^2+^ release in GD3-1 was abolished when pretreated with CPA for 30 min (Fig. 4e). This data suggests an overfilling of ER Ca^2+^ stores in iPSCs-derived neural cells from nGD.

### An increase in UPR and ER stress is observed in NPCs and iPSCs-derived neuronal cell models from neuronopathic GD patients

**Figure 5:**
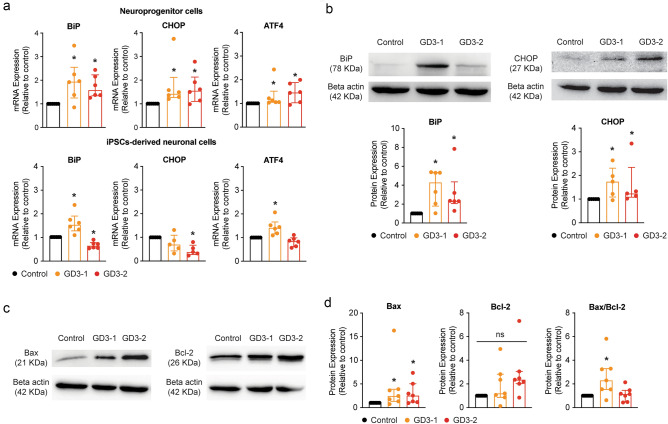
Observation of ER stress and UPR activation in iPSCs-derived neuronal cells from GD. (**a**) (Top) Bar graphs representing the relative mRNA expression levels of BiP (left, Kruskal–Wallis test: H(2) = 8.20, *p* = 0.02 for Control vs. GD3-1, *p* = 0.03 for Control vs. GD3-2, n = 6 technical replicates), CHOP (middle, Kruskal–Wallis test: H(2) = 8.24, *p* < 0.01 for Control vs. GD3-1, *p* = 0.02 for Control vs. GD3-2, n = 6 technical replicates) and ATF4 (right, Kruskal–Wallis test: H(2) = 8.20, *p* = 0.02 for Control vs. GD3-1, *p* = 0.01 for Control vs. GD3-2, n = 6 technical replicates) in NPCs from control, GD3-1 and GD3-2 samples. (Bottom) Bar graphs representing the relative mRNA expression levels of BiP (left, Kruskal–Wallis test: H(2) = 15.73, *p* = 0.04 for Control vs. GD3-1, *p* = 0.04 for Control vs. GD3-2, n = 6 technical replicates), CHOP (middle, Kruskal–Wallis test: H(2) = 7.55, *p* = 0.11 for Control vs. GD3-1, *p* < 0.01 for Control vs. GD3-2, n = 5 technical replicates) and ATF4 (right, Kruskal–Wallis test: H(2) = 12.75, *p* = 0.02 for Control vs. GD3-1, *p* = 0.21 for Control vs. GD3-2, n = 6 technical replicates) in iPSCs-derived neuronal cells from control, GD3-1 and GD3-2 samples. (**b**) (Top) Representative cropped western blot images of GBA1 and CHOP from iPSCs-derived neuronal cells from control, GD3-1 and GD3-2 samples. (Bottom) Bar graphs summarizing of the relative normalized BiP (Kruskal–Wallis test: H(2) = 11.9, *p* < 0.01 for Control vs. GD3-1, *p* < 0.01 for Control vs. GD3-2, n = 6 technical replicates) and CHOP (Kruskal–Wallis test: H(2) = 6.24, *p* = 0.04 for Control vs. GD3-1, *p* = 0.03 for Control vs. GD3-2, n = 5 technical replicates) protein expression in GD3-1 and GD3-2 compared to control group. (**c**) Representative cropped western blot images of Bax (left) and Bcl-2 (right) from iPSCs-derived neuronal cells from control, GD3-1 and GD3-2 samples. (**d**) Bar graphs summarizing of the relative normalized Bax (Kruskal–Wallis test: H(2) = 7.09, *p* = 0.02 for Control vs. GD3-1, *p* = 0.03 for Control vs. GD3-2, n = 7 technical replicates) , Bcl-2 (Kruskal–Wallis test: H(2) = 5.46, *p* = 0.06, n = 7 technical replicates) and the ratio of Bax/Bcl-2 (Kruskal–Wallis test: H(2) = 7.93, *p* < 0.01 for Control vs. GD3-1, *p* = 0.36 for Control vs. GD3-2, n = 7 technical replicates) protein expression in GD3-1 and GD3-2 compared to control group. All replicate western blot images are included in the supplementary information file.

To evaluate the consequences of an increased in ER calcium levels, we investigate one of the key mechanisms involving ER stress and UPR activation^31^. It has been demonstrated that a GBA1 mutation leads to protein misfolding, resulting in its accumulation in the ER. This accumulation, in turn, activates the UPR, which can potentially lead to either the alleviation of ER stress or the initiation of apoptosis^11^. Given that ER stress is often a persistent process, we decided to examine UPR activation in both NPC and iPSCs-derived neuronal cells. We perform RT-qPCR analysis of *BiP*, *CHOP*, and *ATF4*, which are prominent UPR activation markers as previously described^19^. We initially assessed the mRNA expression of UPR activation markers, including BiP, CHOP, and ATF4, in both NPC and iPSCs-derived neuronal cells. Figure 5A (top) shows a significant increase in mRNA expression of BiP, CHOP, and ATF4 in both NPCs from GD3-1 and GD3-2. Meanwhile, a significant increase in BiP and ATF4 mRNA was observed in iPSCs-derived neuronal cells from nGD, but only in GD3-1 and not GD3-2 (Fig. 5a, bottom). The analysis of CHOP also revealed a decrease in mRNA expression in both GD brain cells compared to the control (Fig. 5a, bottom). Subsequently, we measured the protein levels of downstream consequences of ER stress and UPR in iPSCs-derived neuronal cells. Figure 5b reveals a significantly increase in BiP and CHOP protein levels in GD3-1 and GD3-2 compared to control group.

The activation of the UPR through the BiP, CHOP, and ATF4 cascade can lead to the initiation of downstream apoptosis pathways by increasing the ratio of pro-apoptotic to anti-apoptotic BAX/BCL-2^32,33^. To determine the activation of apoptosis from the ER stress, we look at the ratio of expression of BAX and BCL-2. In the experiments, a significant increase in the BAX/BCL-2 ratio was observed in GD3-1, while GD3-2 showed a significant increase in BAX protein. However, there was also a significant increase in Bcl-2 to counteract the BAX/BCL-2 ratio in GD3-2 (Fig. 5c,d). These results suggest the presence of ER stress in GD neurons and its activation of an apoptosis pathway in iPSCs-derived neuronal cells from nGD.

### An increase in apoptosis in iPSC-derived neuronal cells model from neuronopathic GD patients

**Figure 6:**
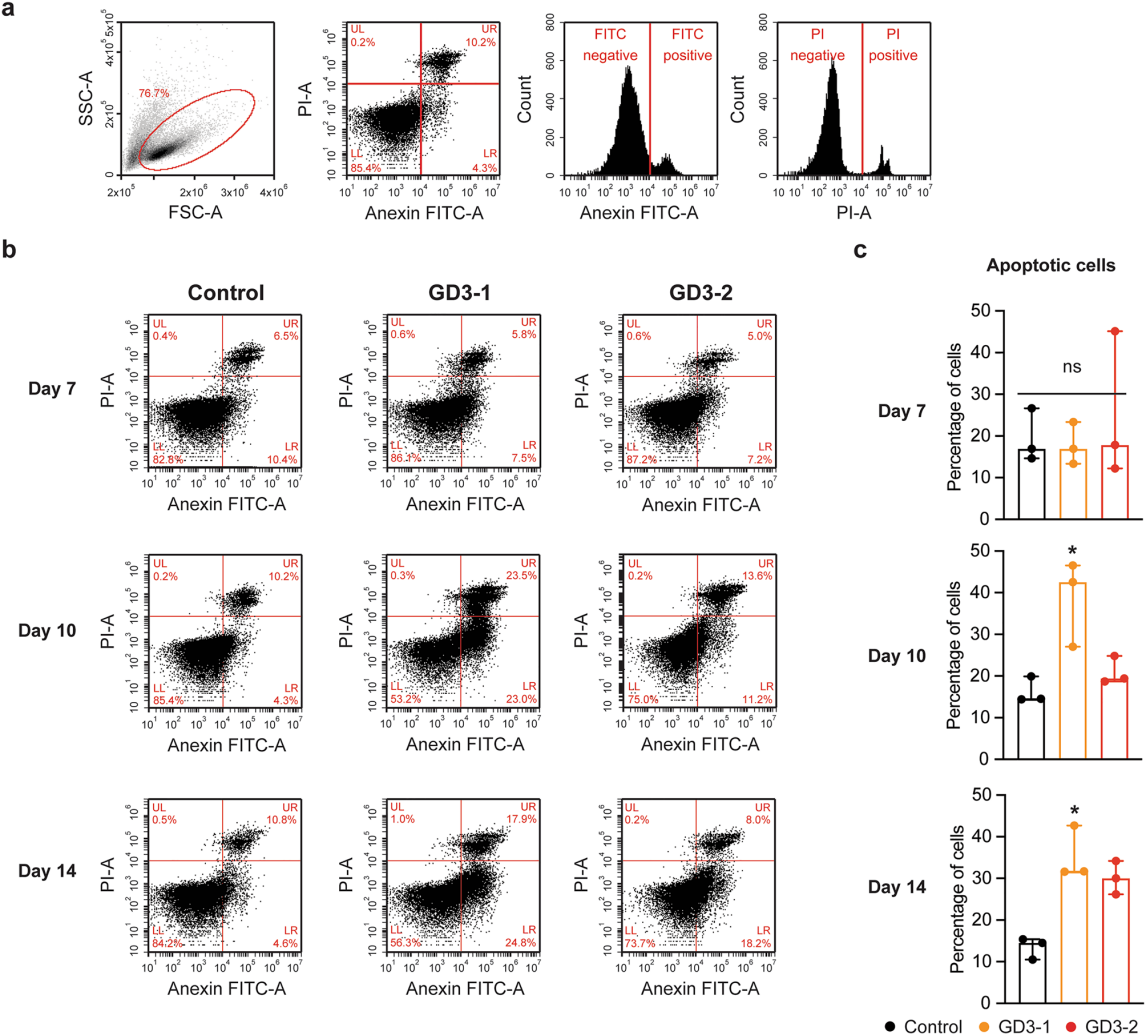
Different in neuronal apoptosis in iPSCs-derived neuronal cell from GD patients. (**a**) Scatter plots represent a complete gating strategy in the flow cytometry analysis. (**b**) Scatter plots represent the intensities of PI and AnnexinV-FITC fluorescent at day 7, 10 and 14 of neuronal differentiation in control, GD3-1 and GD3-2 groups. (**c**) Bar graphs represent the percentage of apoptotic cells at day 7 (Kruskal–Wallis test: H(2) = 0.16, *p* = 0.95, n = 3 technical replicates), day 10 (Kruskal–Wallis test: H(2) = 5.97, *p* = 0.02 for Control vs. GD3-1, *p* = 0.46 for Control vs. GD3-2, n = 3 technical replicates) and day 14 (Kruskal–Wallis test: H(2) = 5.96, *p* = 0.02 for Control vs. GD3-1, *p* = 0.10 for Control vs. GD3-2, n = 3 technical replicates) of neuronal differentiation.

To assess the activation of the UPR and ER stress leading to apoptosis, we conducted flow cytometry analysis to identify apoptotic cells marked by double positivity for Annexin V and propidium iodide (PI). Annexin V binds to phosphatidylserine, which is typically exposed during apoptosis, while PI is an impermeable dye that can be positive in cells with compromised membrane permeability. To determine the temporal progression of apoptosis, flow cytometry was performed on days 7, 10, and 14 of the differentiation process. The gating of apoptotic cells was achieved using unstained and single-stained cells (Fig. 6a). The flow cytometry results revealed a significant increase in the number of both early and late apoptotic cells (FITC positive cells) on day 10 and day 14 in the GD3-1 group compared to the control while GD3-2 shows a trend to increase in the number of apoptotic cells on day 14 (Fig. 6b,c). Notably, there is a difference in the pattern of apoptotic cell (both early and late apoptotic) increase between GD3-1 and GD3-2. GD3-1 appears to exhibit an increase in apoptotic cell death as early as day 10 of differentiation. In contrast, GD3-2 shows this increase later, on day 14 of differentiation. This suggests a different susceptibility to apoptosis between GD3-1 and GD3-2.

### Analysis of modifying genes related to Gaucher’s disease and unfolded protein response in neuronal cell models derived from iPSCs of neuronopathic GD patients

While both GD3-1 and GD3-2 carry the known similar *GBA1* variants, there exists a difference in susceptibility to neuronal death between these two cell lines. One possible explanation for this difference lies in the presence of modifying factor genes. Therefore, we conducted WES on genomic DNA extracted from the peripheral blood of our donors. We compiled lists of known genes associated with the ER stress response and potential modifying genes, which included prosaposin, Scavenger receptor class B member, and lysosomal integral membrane protein 2, for comparison (summarized in Supplementary Table 2,^34,35^). Various variants in genes related to the ER stress response and potential modifying genes in GD were identified between GD3-1 and GD3-2 and are summarized in Supplementary Table 3. Upon closer analysis, only one candidate emerged as a potential modifying factor distinguishing the two patients: the rs2228570 polymorphism of *VDR* (Vitamin D receptor). It is also noteworthy that variants in *BCL-2*, *GBA3*, T*MEM175*, and *TNFRSF11B* were observed in both patients.

## Discussion

**Figure 7:**
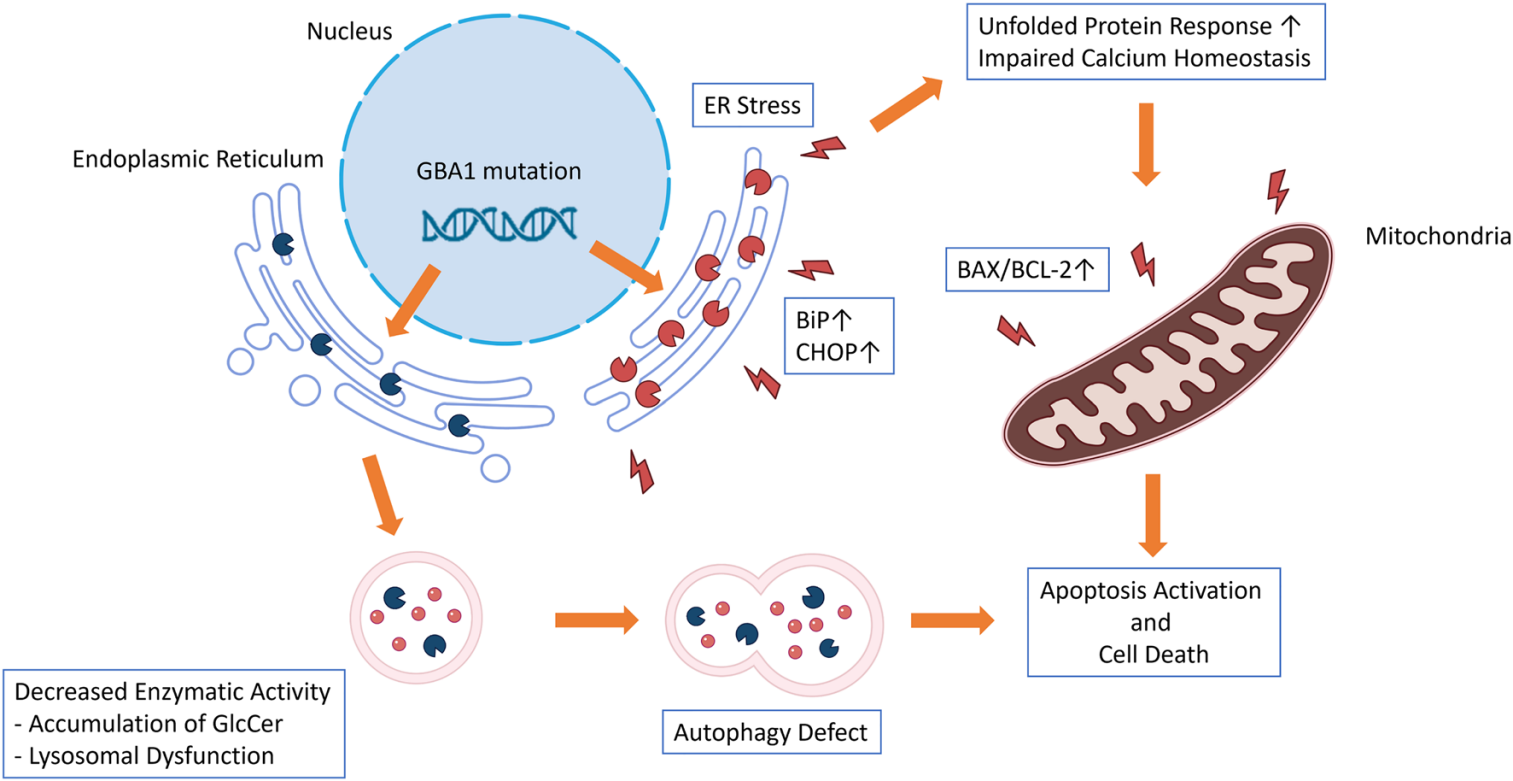
Mechanism of ER stress and unfolded protein response induced cellular injury in iPSCs-derived neuronal cell from GD patient. A schematic showing GBA1 mutation causing ER stress and triggering unfolded protein response leading to cellular death in iPSCs-derived neuronal cells from GD patients.

Gaucher disease is a rare genetic disorder that has been extensively studied, yet there is still a limited understanding of the mechanisms leading to neurological deficits in patients with this condition. Additionally, it remains puzzling why individuals with the same mutation can exhibit varying disease manifestations. Furthermore, there is no clear evidence supporting the idea that current treatments are designed to address the neurological form of Gaucher disease, resulting in a significant gap in care for those with neurological involvement. In the present study, we used our recently established iPSCs-derived neuronal cell lines from two individuals with neuronopathic Gaucher disease (nGD) patients. When compared to iPSCs-derived neuronal cells from healthy individuals, we have identified evidence indicating an increase in ER stress in the iPSCs-derived neuronal cells of nGD patients. This elevated ER stress leads to the downstream activation of the UPR, ultimately triggering the apoptosis pathway and resulting in an increase in neuronal death (Fig. 7).

Induced pluripotent stem cells (iPSCs) serve as a potent cellular model for investigating the pathogenesis of severe diseases^36^ due to their ability to replicate nearly all aspects of the patient, particularly their genetic profile. This quality makes iPSCs particularly valuable in the study of rare genetic diseases, such as Lysosomal Storage Diseases (LSDs). In our study, we performed an experiment using the two most recently generated iPSCs lines from type-3 Gaucher disease (GD) patients^24,25^. These cell lines, derived from type-3 GD patients, exhibited hallmark GD phenotypes, including a reduction in GBA protein expression (Fig. 3a). This reduction led to an overall decrease in glucocerebrosidase enzyme activity, as demonstrated in both neural progenitor cells (NPC) and brain cells during differentiation (Fig. 3b). Furthermore, in addition to the reduced enzymatic activity, we observed an increase in the lysosome count as well as heightened autophagic activity in these iPSCs-derived neuronal cells (Fig. 3c,d). These findings align with various phenotypic observations made in the peripheral blood of GD patients and in the brains of mouse models of GD^37–39^. After differentiation, we observed a decrease in the mRNA expression of GAD67, a marker for interneurons, in both nGD cell lines (GD3-1, GD3-2; Fig. 2b). These findings indicate a reduced proportion of interneurons in the iPSCs-derived neuronal cells in nGD, which aligns with observations in several neurodegenerative diseases such as Batten disease^40^ and infantile neuronal ceroid lipofuscinosis, a fatal hereditary lysosomal storage disorder^41^. These reports collectively suggest a higher susceptibility to neuronal injury in interneurons. However, further studies are required to elucidate why interneurons exhibit this heightened susceptibility.

One of the potential pathogenetic mechanisms in GD is the impairment of autophagy. This impairment has been observed in neurons affected by GD, suggesting a possible role in the neuronal death associated with the condition. Additionally, studies have suggested that increased in ER stress can activate autophagy pathways^42^. In our study, we observed an increase in the number of lysosomes and autolysosomes in GD-affected neurons using both SiR-lysosome and acridine orange dye, as well as though western blot analysis of LAMP1 and LC3I/II proteins. We speculate that the upregulation of autophagy pathways and impairment of autophagic flux may also contribute to the pathophysiology of GD. However, it remains unclear whether lysosomal dysfunction also plays a role in lysosomal acidification in GD, as reported in one neuronal model of GD^43^.

Our finding further indicates an elevation in the expression of UPR genes and proteins like BiP, CHOP, and ATF4, suggesting an increase in ER stress in the iPSCs-derived neuronal cells of individuals with type-3 Gaucher disease (GD). This likely results from the accumulation of aberrant GCase in the ER. Several *GBA1* pathogenic variants, including L444P and N370S, are missense mutations that lead to abnormal amino acid sequences, ultimately causing misfolding of GCase during post-translation processes^44^. While this process is common in many diseases involving misfolded proteins, the presence of ER stress and UPR in GD is still controversial. Some reports have found no increase in UPR markers or evidence of ER stress in conduritol-β-epoxide (CBE) treated mouse primary neurons and primary astrocytes culture^23^, while others have detected the presence of ER stress and UPR in fibroblasts of GD patients and in the brain of mouse models of GD^19–22^. Furthermore, our research revealed an increase in apoptosis activation due to the elevated ratio of BAX/BCL-2, which is known to be increased subsequent to CHOP overactivation and a higher number of apoptotic cells in the culture of type-3 GD neurons compared to the normal control. These findings support the existence of ER stress and activation of UPR in the GD neuronal model, indicating that these processes eventually lead to apoptosis activation resulting in increased neuronal death. This suggests a possible contribution to the neurological symptoms associated with nGD. During our investigation of UPR in GD neurons, we observed a discrepancy between CHOP mRNA and protein expression. Apart from the mismatching in mRNA and protein expressions due to possible post-transcriptional regulation the cells, we hypothesized that, at later stages, excessive activation of apoptosis signaling from UPR activation caused the downregulation of CHOP gene expression and that the effect at mRNA level is still not reflected at the protein. A similar phenomenon was found in the excessive expression of the human homolog of Drosophila tribbles (TRB3), which could result in the decrease in CHOP mRNA levels^45^. TRB3 is a downstream protein to CHOP in the UPR-apoptosis activation pathway, with the role of further driving apoptosis signaling^46^. Other than inducing apoptosis, TRB3 also fine-tunes the pivot of cell survival by regulating its activators, such as ATF4 and CHOP^47^. It is thus speculated that TRB3 is the deciding factor in the apoptotic fate of the cells^45^. However, the exact effect of TRB3 in the context of GD remains to be studied.

The heterogeneity in neurological manifestations and disease progression is observed among individuals with neuronopathic Gaucher disease, even when they carry the same *GBA1* variant^48^. Our findings reveal variations in the levels of increased ER stress and activation of the UPR, leading to apoptosis in iPSCs-derived neuronal cells from 2 nGD patients. One potential explanation for this heterogeneity is the presence of modifying genes, as indicated by several previous reports in GD patients as well as in the mouse model of GD^34,49^. In this study, we compare the known modifying genes of ER stress and GD between 2 samples, in hope to find the possible explanation for the differences in their susceptibilities. Only one candidate, VDR polymorphism rs2228570 was found in GD3-1. This polymorphism has been reported to be associated with PD. However, the information regarding rs2228570 polymorphism is still largely unknown, though it is thought that the polymorphism causes reduction in intestinal vitamin D absorption, leading to the alteration of various neurotropic factors production, eventually causing susceptibility to PD development^50,51^. On the other hand, we also found the similarities of genetic polymorphisms between the 2 patients, even though they are not related (summarized as shown in Supplementary Table 3). The variant of *GBA3* is proposed to diminish residual cytoplasmic GCase activity of GBA3 which could reduce GlcCer load in the cells of GD type-2 and GD type-3^34^. However, the specific mutation found in our case is thought to be the result of an evolutionary effect in humans. TMEM175 is a crucial member of lysosomal K^+^ channel playing its role in stabilizing lysosomal pH and maintaining activities of lysosomal enzymes. Its deficiency results in impairment of autophagy and mitophagy process^52^. TNFRSF11B on the other hand is reported to have protective effect on maintaining bone mineral density, its impairment could be playing a part in bone manifestations of GD^53^. Our result might indicate that these indicated genes play a part in the symptoms of the two GD type-3 patients. However, future studies with a larger cohort are needed to confirm the actual role of these genes in GD patients. As well as the specific genetic modification by CRISPR/Cas9 technology in iPSCs to investigate the effects of altering these single nucleotide polymorphisms could be provide the information about the actual role of these SNPs in the nGD pathogenesis. Although, this 2D iPSCs-derived neuronal cell models exhibit nGD pathogenesis, it is also a limitation of this model that exhibit less recapitulating complex neuronal network and multicellular microenvironment when compare to 3D organoid model^54,55^. Therefore, the impact of ER-stress and unfolded protein response in the more complex networking models remains an open question.

## Conclusions

In conclusion, these results demonstrate neuronopathic Gaucher disease phenotypes in two neurological GD-models derived from type-3 GD patients. These data are consistent with pathological findings related to Gaucher disease, including a decrease in GCase protein levels and enzymatic activities, an increase in lysosomal content and its autophagic activities, as well as evidence of ER stress and activation of the UPR. These findings further contribute to our understanding of the pathogenesis of neuronopathic Gaucher disease.

### Supplementary Information

Supplementary Video 1.

Supplementary Video 2.

Supplementary Video 3.

Supplementary Video 4.

Supplementary Video 5.

Supplementary Video 6.

Supplementary Information 1.

Supplementary Information 2.

Supplementary Information 3.

## Data Availability

All data presented in this study are available within the manuscript and supplementary material.

## Abbreviations

ATF6: Activating transcription factor 6
BiP: Binding immunoglobulin protein
CHOP: C/EBP homology protein
ER: Endoplasmic reticulum
Gcase: Glucocerebrosidase
GD: Gaucher disease
HBSS: Hanks’ balanced salt solution
iPSCs: Induced pluripotent stem cells
IRE1: Inositol-requiring transmembrane kinase/endoribonuclease 1
LSDs: Lysosomal storage diseases
nGD: Neuronopathic Gaucher disease
NPCs: Neural progenitor cells
PERK: Double-stranded RNA-dependent protein kinase-like eukaryotic initiation factor 2α kinase
UPR: Unfolded protein response
