# Supplementary material for: An increase in ER stress and unfolded protein response in iPSCs-derived neuronal cells from neuronopathic Gaucher disease patients
Source: Sci Rep. 2024 Apr 22;14:9177. doi: 10.1038/s41598-024-59834-6 (PMC11035702; doi:10.1038/s41598-024-59834-6)
Supplement: Supplementary file 8 — Supplementary Information 2. [file 41598_2024_59834_MOESM8_ESM.pdf]

Supplementary Table 1

Genes	Forward primer sequences	Reverse primer sequences
NeuN	5’-TCGTAGAGGGACGGAAAATTGA-3′	5’-GCCGTTGGTGTAGGGGTTC-3′
VGLUT1	5’-TTGATGAACTGCGGAGGCTT-3’	5’-GTTGAACCCAGAGATGGCGA-3’
GAD67	5’-GGGAACTAGCGAGAACGAGG-3’	5’-GGGCGCAGGTTAGTGGTATT-3’
GFAP	5’-ATCCACGAGGAGGAGGTTCG-3’	5’-CATGTTGCTGGACGCCATTG-3’
CHOP	5′-AGCGACAGAGCCAAAATCAG-3′	5′-TCTGCTTTCAGGTGTGGTGA-3′
BiP	5′-CATCAAGTTCTTGCCGTTCA-3′	5′-ATGTCTTTGTTTGCCCACCT-3′
ATF4	5′-GTTCTCCAGCGACAAGGCTA-3′	5′-ATCCTGCTTGCTGTTGTTGG-3′
GAPDH	5’-GAAATCCCATCACCATCTTCC-3’	5’- AAATGAGCCCCAGCC TTCTC -3’

List of the primers used for real time PCR

Supplementary Table 2

ABCE1	ER STRESS ASSOCIATED GENES	GPR84	ER STRESS ASSOCIATED GENES
AHSA1	ER STRESS ASSOCIATED GENES	GRN	GD MODIFIERS
ALDH1L2	ER STRESS ASSOCIATED GENES	GRPEL1	ER STRESS ASSOCIATED GENES
ALOX5AP	ER STRESS ASSOCIATED GENES	HERPUD1	ER STRESS ASSOCIATED GENES
ALPK2	ER STRESS ASSOCIATED GENES	HPDL	ER STRESS ASSOCIATED GENES
ASF1B	ER STRESS ASSOCIATED GENES	HSP90AA1	ER STRESS ASSOCIATED GENES
ASS1	ER STRESS ASSOCIATED GENES	HSP90AB1	ER STRESS ASSOCIATED GENES
BAG1	ER STRESS ASSOCIATED GENES	HSP90B1	ER STRESS ASSOCIATED GENES
BAG2	ER STRESS ASSOCIATED GENES	HSPA1A	ER STRESS ASSOCIATED GENES
BAT5	ER STRESS ASSOCIATED GENES	HSPA2	ER STRESS ASSOCIATED GENES
BIN1	GD MODIFIERS	HSPA4	ER STRESS ASSOCIATED GENES
CALR	ER STRESS ASSOCIATED GENES	HSPA4L	ER STRESS ASSOCIATED GENES
CANX	ER STRESS ASSOCIATED GENES	HSPA8	ER STRESS ASSOCIATED GENES
CCT2	ER STRESS ASSOCIATED GENES	HSPB1	ER STRESS ASSOCIATED GENES
CCT6A	ER STRESS ASSOCIATED GENES	HSPE1	ER STRESS ASSOCIATED GENES
CCT8	ER STRESS ASSOCIATED GENES	HSPH1	ER STRESS ASSOCIATED GENES
CDRT4	ER STRESS ASSOCIATED GENES	HYOU1	ER STRESS ASSOCIATED GENES
CHAF1A	ER STRESS ASSOCIATED GENES	IL-6	GD MODIFIERS
CLGN	ER STRESS ASSOCIATED GENES	INHBE	ER STRESS ASSOCIATED GENES
COPA	ER STRESS ASSOCIATED GENES	ITCH	GD MODIFIERS
CREB3L2	ER STRESS ASSOCIATED GENES	KISS1R	ER STRESS ASSOCIATED GENES
CREB3L4	ER STRESS ASSOCIATED GENES	MSH6	GD MODIFIERS
DDIT3	ER STRESS ASSOCIATED GENES	MTX1	GD MODIFIERS
DERL1	ER STRESS ASSOCIATED GENES	NFE2L1	ER STRESS ASSOCIATED GENES
DERL2	ER STRESS ASSOCIATED GENES	P4HB	ER STRESS ASSOCIATED GENES

DERL3	ER STRESS ASSOCIATED GENES	PCK2	ER STRESS ASSOCIATED GENES
DNAJA1	ER STRESS ASSOCIATED GENES	PHGDH	ER STRESS ASSOCIATED GENES
DNAJA4	ER STRESS ASSOCIATED GENES	PSAP	GD MODIFIERS
DNAJB1	ER STRESS ASSOCIATED GENES	PSAT1	ER STRESS ASSOCIATED GENES
DNAJB11	ER STRESS ASSOCIATED GENES	RPS6KA2	ER STRESS ASSOCIATED GENES
DNAJB5	ER STRESS ASSOCIATED GENES	SCARB2	GD MODIFIERS
DNAJB6	ER STRESS ASSOCIATED GENES	SCO1	ER STRESS ASSOCIATED GENES
DNAJB9	ER STRESS ASSOCIATED GENES	SEC63	ER STRESS ASSOCIATED GENES
DNAJC1	ER STRESS ASSOCIATED GENES	SELS	ER STRESS ASSOCIATED GENES
DNAJC3	ER STRESS ASSOCIATED GENES	SERPINH1	ER STRESS ASSOCIATED GENES
DNAJC6	ER STRESS ASSOCIATED GENES	SLC1A5	ER STRESS ASSOCIATED GENES
DNAJC9	ER STRESS ASSOCIATED GENES	SLC3A2	ER STRESS ASSOCIATED GENES
EDEM1	ER STRESS ASSOCIATED GENES	SLC7A5	ER STRESS ASSOCIATED GENES
EIF2A3	ER STRESS ASSOCIATED GENES	SNTB1	ER STRESS ASSOCIATED GENES
EML2	ER STRESS ASSOCIATED GENES	TFEB	GD MODIFIERS
ERN1	ER STRESS ASSOCIATED GENES	TIMM8A	ER STRESS ASSOCIATED GENES
ERP44	ER STRESS ASSOCIATED GENES	TMEM175	GD MODIFIERS
ESR1	GD MODIFIERS	TNFRSF11B	GD MODIFIERS
FKBP14	ER STRESS ASSOCIATED GENES	TNF-α	GD MODIFIERS
FKBP5	ER STRESS ASSOCIATED GENES	TSLP	ER STRESS ASSOCIATED GENES
GBA2	GD MODIFIERS	UGCG	GD MODIFIERS
GBA3	GD MODIFIERS	VDR	GD MODIFIERS
GDF11	ER STRESS ASSOCIATED GENES	VLDLR	ER STRESS ASSOCIATED GENES

List of known genes associated with ER stress and modifying genes in Gaucher disease, which were used in this study.

Supplementary Table 3

GD3-1

Gene Name	Feature ID	dbSNP ID	Nucleotide Change (HGVS.c)	Amino Acid Change (HGVS.p)	Effect	Function
GBA3	NM_001277225.1	rs358231	c.1371A>T	p.Ter457Tyrext*?	Stop loss	Cytosolic β-glucosidase (Pseudogene)
TMEM175	NM_032326.3	rs2290405	c.463-4G>A	-	Splice region variation	Lysosomal K+ channel
VDR	NM_000376.2	rs2228570	c.2T>C	p.Met1Thr	Start loss	Vit-D receptor
TNFRSF11B	NM_002546.3	rs3134046, rs2073618	c.401-5T>C, c.9C>G	- , p.Asn3Lys	Splice region variation, missense variation	Osteoprotegerin

GD3-2

Gene Name	Feature ID	dbSNP ID	Nucleotide Change (HGVS.c)	Amino Acid Change (HGVS.p)	Effect	Function
GBA3	NM_001277225.1	rs358231	c.1371A>T	p.Ter457Tyrext*?	Stop loss	Cytosolic β-glucosidase (Pseudogene)
TMEM175	NM_032326.3	rs2290405	c.463-4G>A	-	Splice region variation	Lysosomal K+ channel
TNFRSF11B	NM_002546.3	rs3134046, rs2073618	c.401-5T>C, c.9C>G	- , p.Asn3Lys	Splice region variation, missense variation	Osteoprotegerin

List of the identified gene variants in GD3-1 and GD3-2 from the selected list as shown in Supplementary Table 2.
