# Supplementary material for: An increase in ER stress and unfolded protein response in iPSCs-derived neuronal cells from neuronopathic Gaucher disease patients
Source: Sci Rep. 2024 Apr 22;14:9177. doi: 10.1038/s41598-024-59834-6 (PMC11035702; doi:10.1038/s41598-024-59834-6)
Supplement: Supplementary file 9 — Supplementary Information 3. [file 41598_2024_59834_MOESM9_ESM.pdf]

GBA1-1

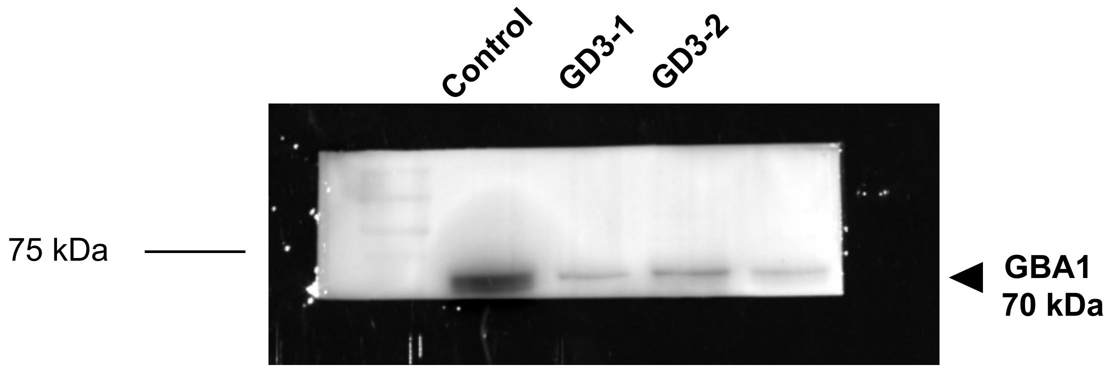

Control
GD3-1
GD3-2
75 kDa
GBA1
70 kDa

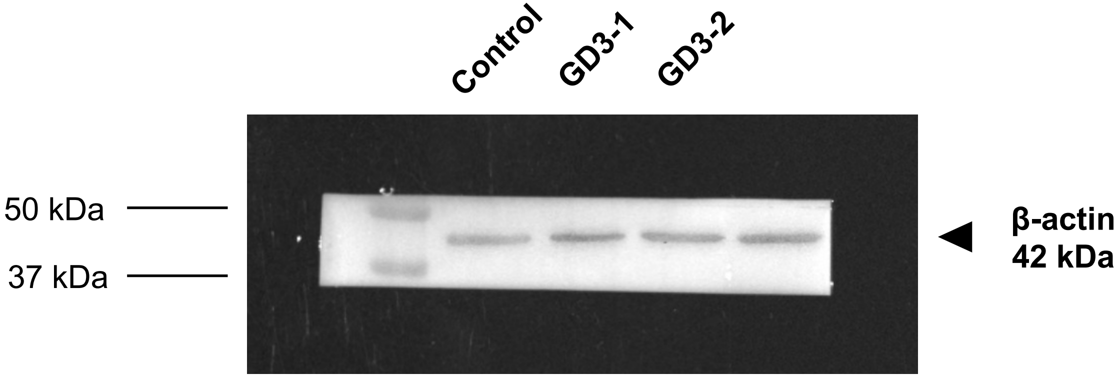

Control
GD3-1
GD3-2
50 kDa
37 kDa
β-actin
42 kDa

GBA1-2

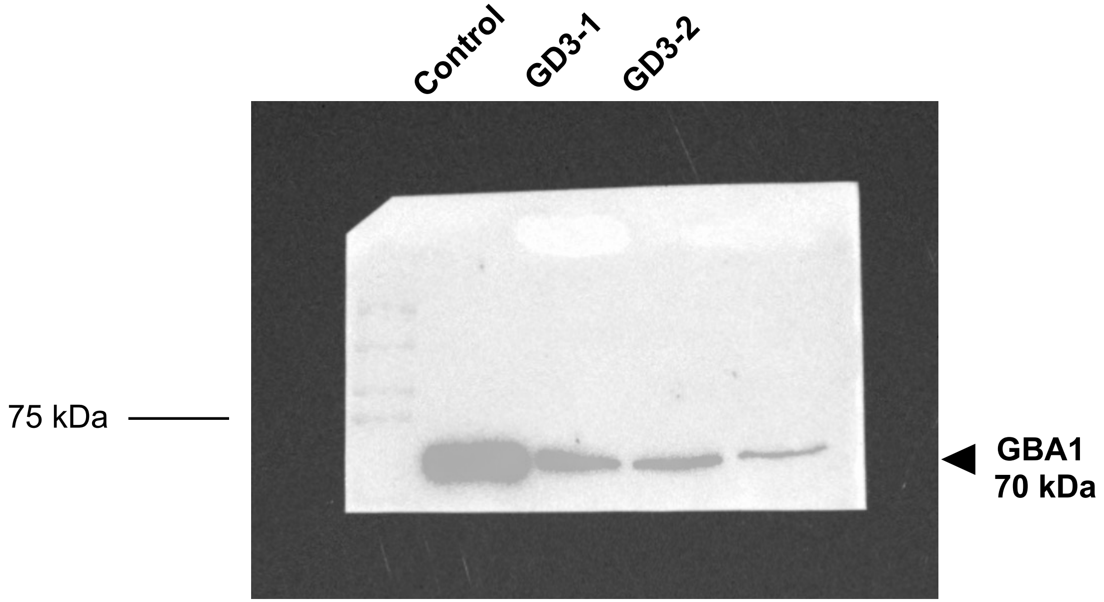
Control
GD3-1
GD3-2
75 kDa
GBA1
70 kDa

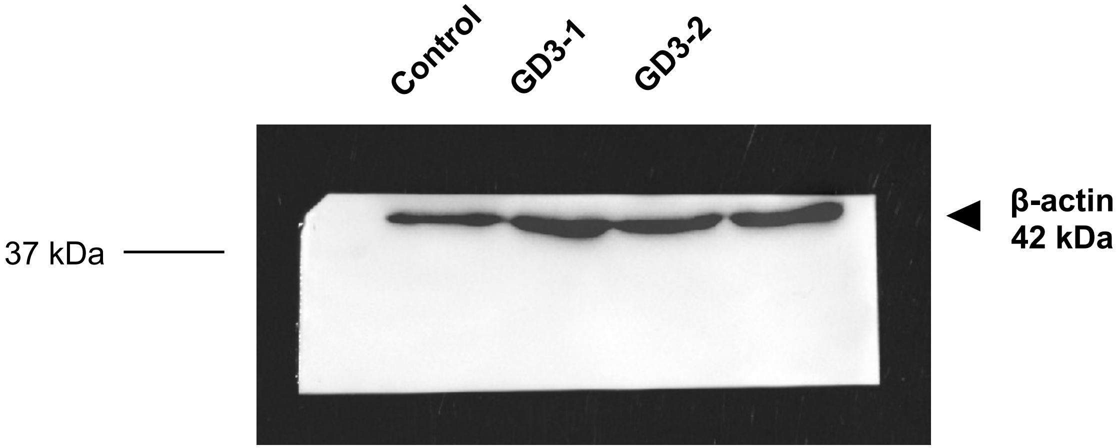
Control
GD3-1
GD3-2
37 kDa
β-actin
42 kDa

GBA1-3

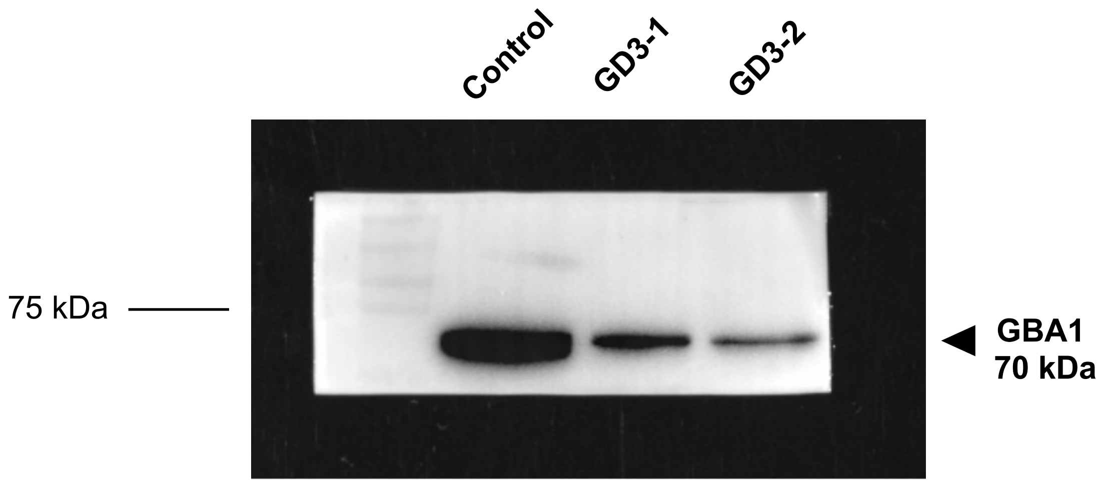
Control
GD3-1
GD3-2
75 kDa
GBA1
70 kDa

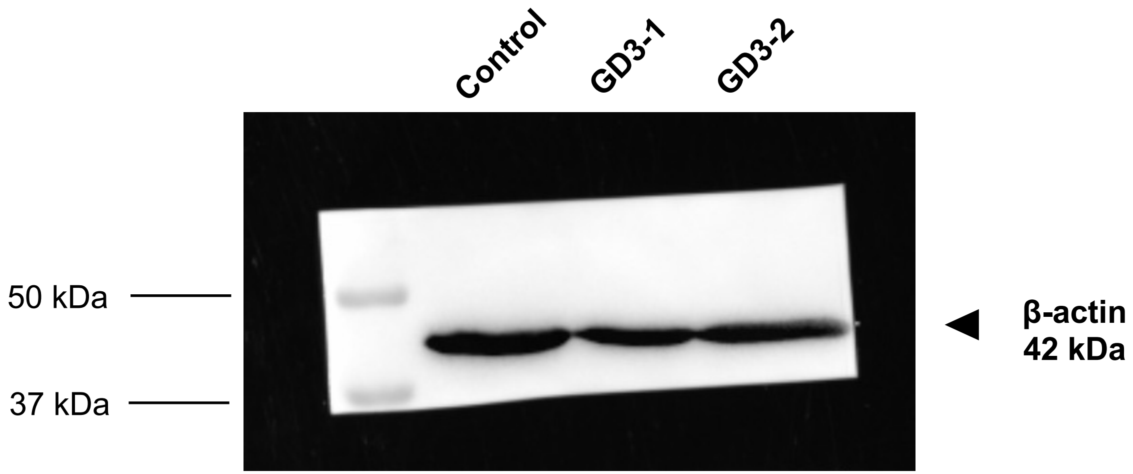
Control
GD3-1
GD3-2
50 kDa
37 kDa
β-actin
42 kDa

GBA1-4

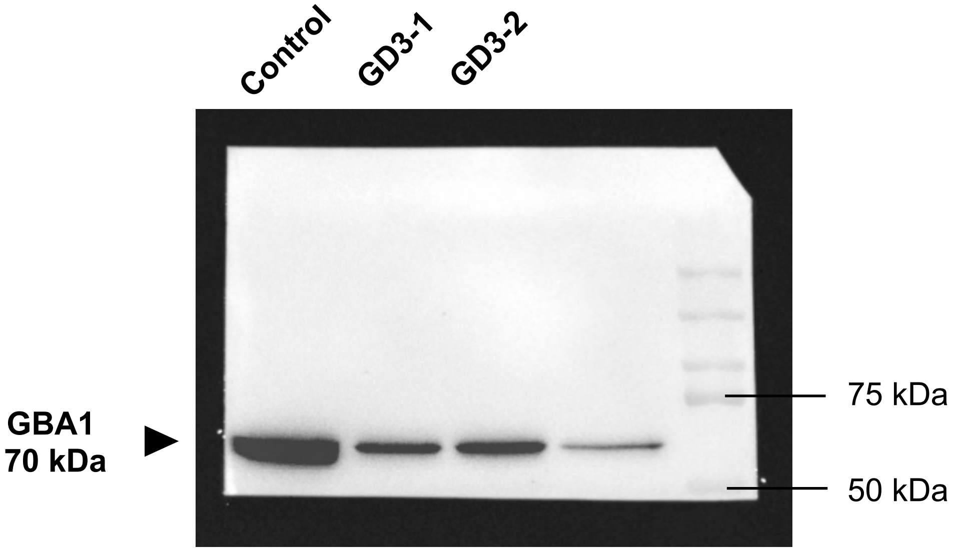
Control
GD3-1
GD3-2
GBA1
70 kDa
75 kDa
50 kDa

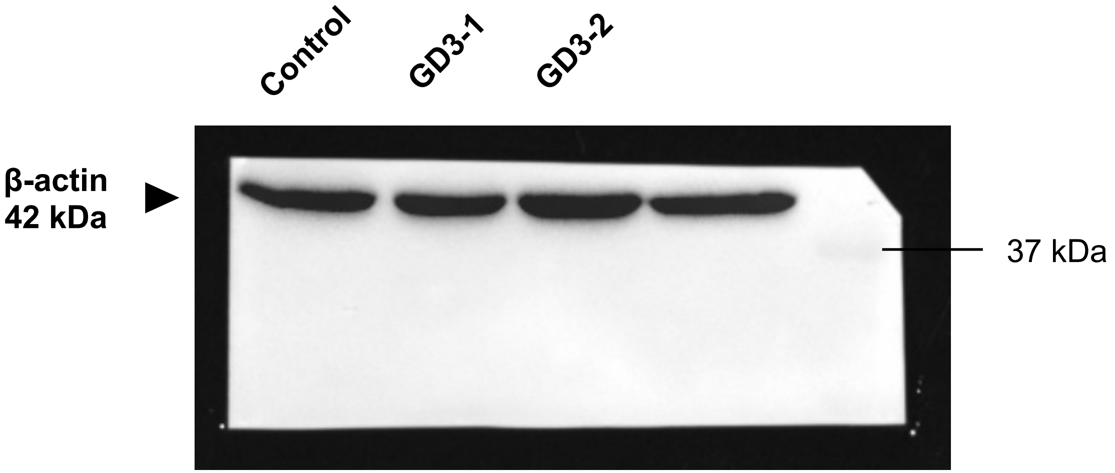
Control
GD3-1
GD3-2
β-actin
42 kDa
37 kDa

GBA1-5

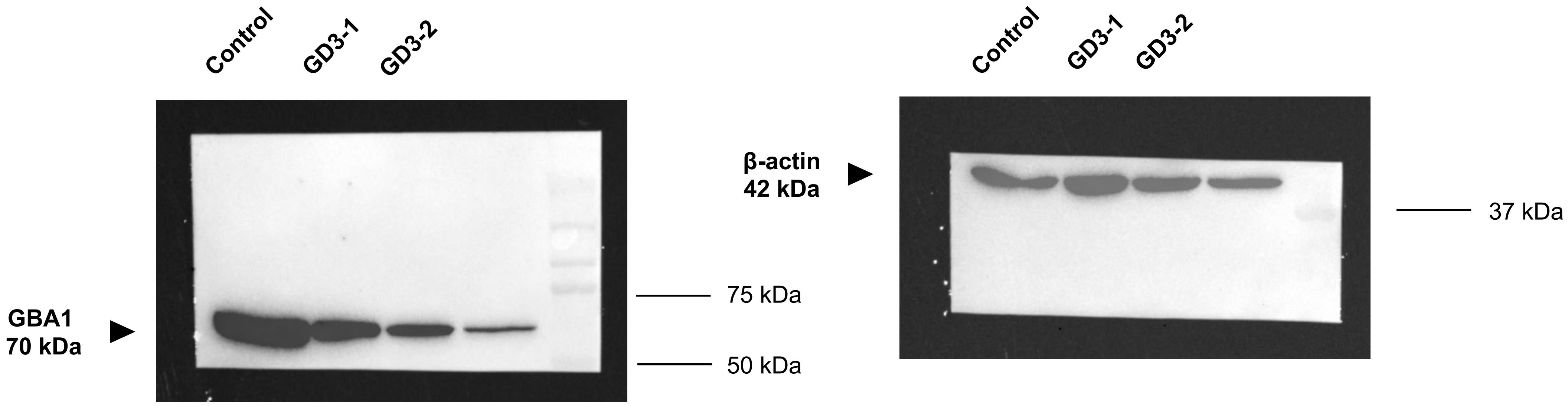
Control
GD3-1
GD3-2
GBA1
70 kDa
75 kDa
50 kDa
Control
GD3-1
GD3-2
β-actin
42 kDa
37 kDa

LAMP1 & LC3

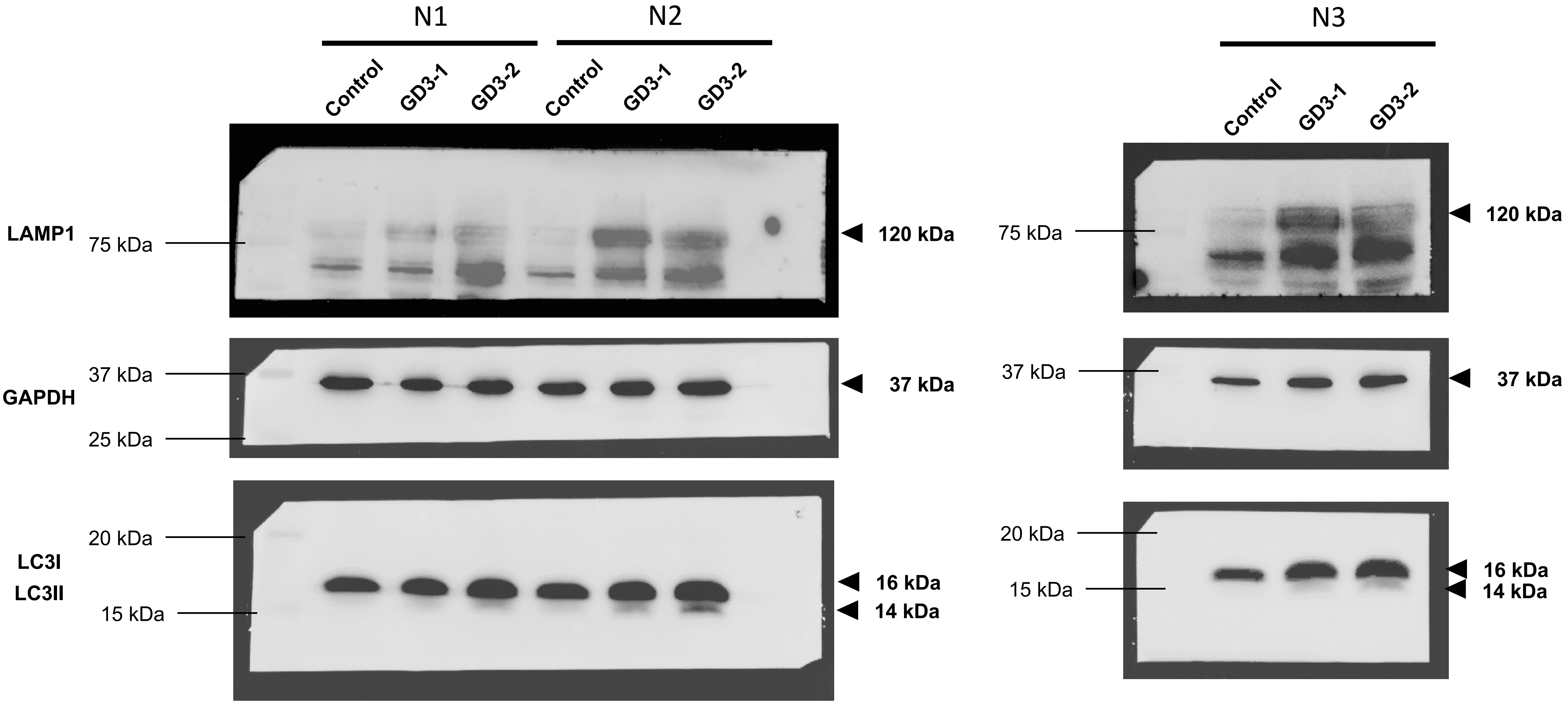
N1
N2
N3
Control
GD3-1
GD3-2
Control
GD3-1
GD3-2
Control
GD3-1
GD3-2
LAMP1
75 kDa
120 kDa
75 kDa
120 kDa
37 kDa
GAPDH
25 kDa
37 kDa
37 kDa
37 kDa
20 kDa
LC3I
LC3II
15 kDa
16 kDa
14 kDa
20 kDa
15 kDa
16 kDa
14 kDa

BiP-1

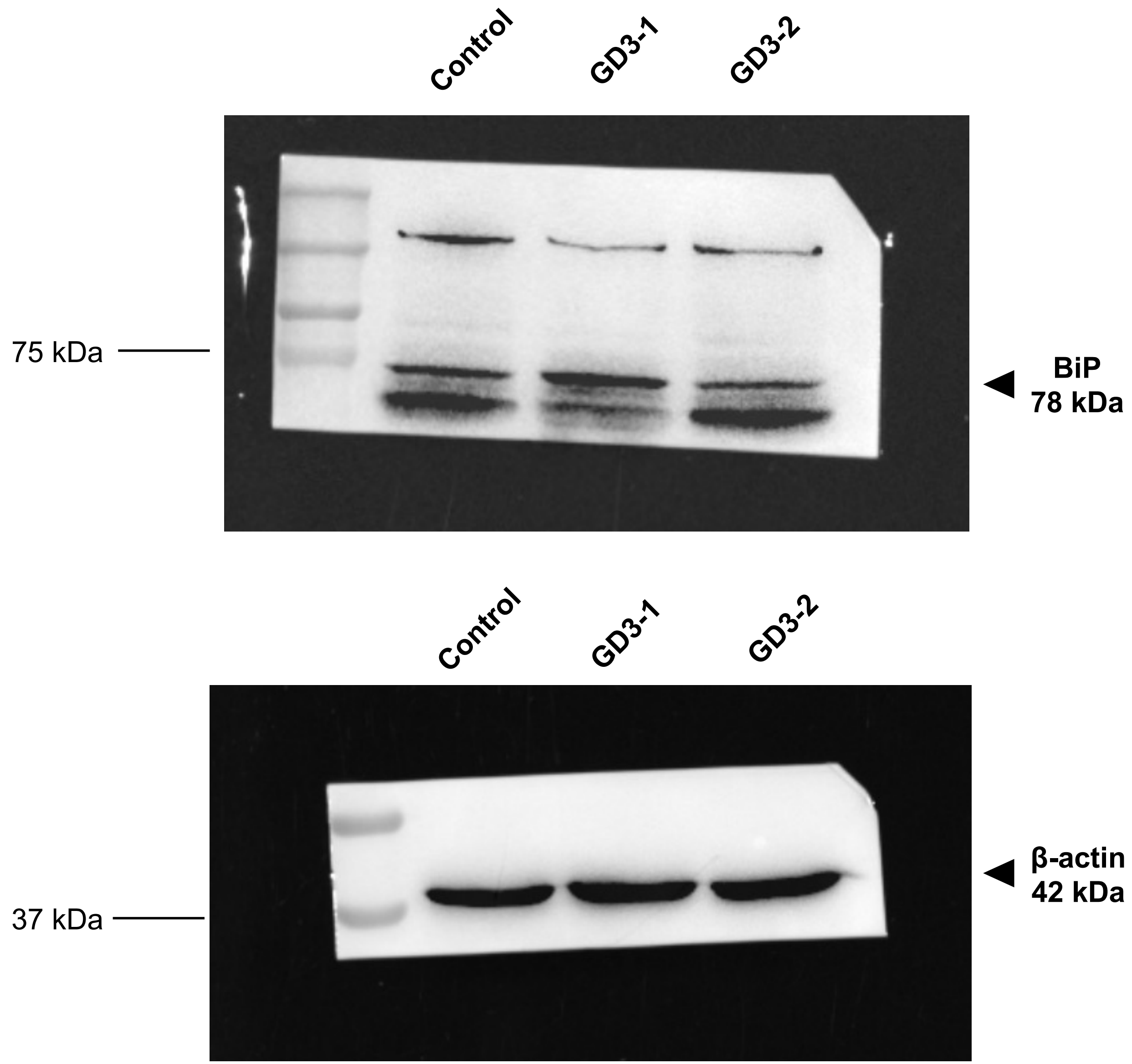
Control
GD3-1
GD3-2
75 kDa
BiP
78 kDa
Control
GD3-1
GD3-2
37 kDa
β-actin
42 kDa

BiP-2

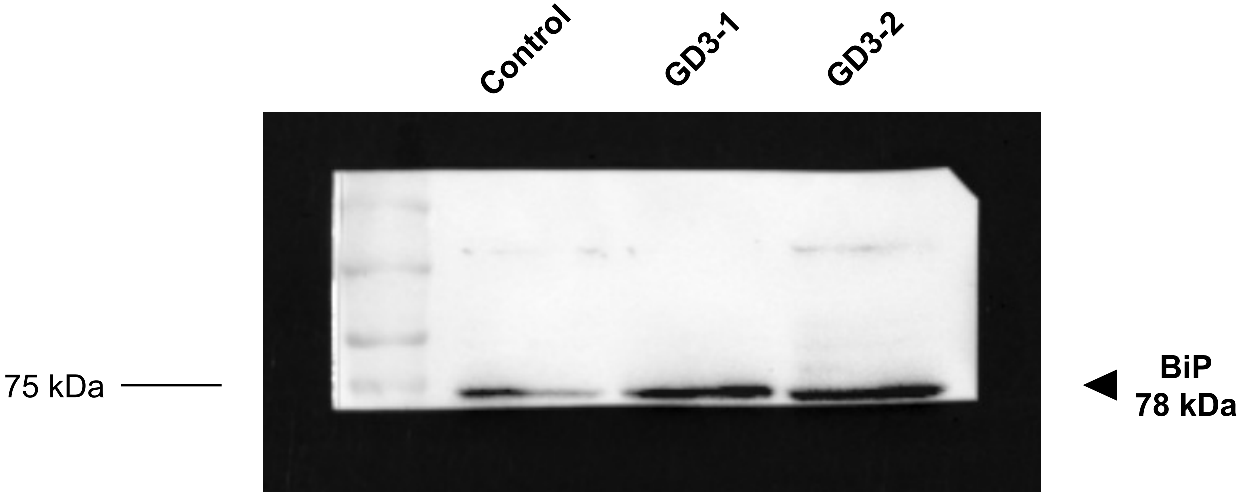
Control
GD3-1
GD3-2
75 kDa
BiP
78 kDa

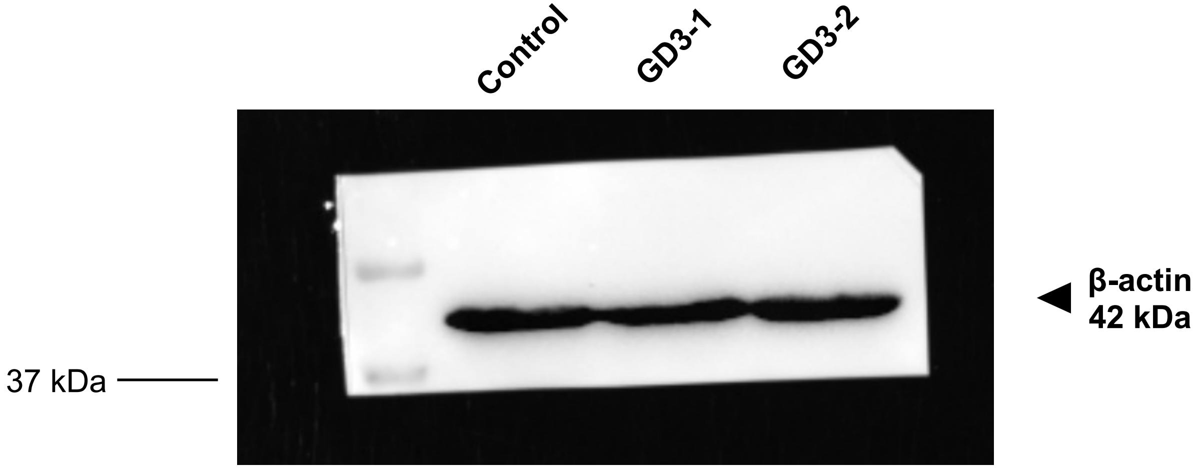
Control
GD3-1
GD3-2
β-actin
42 kDa
37 kDa

BiP-3

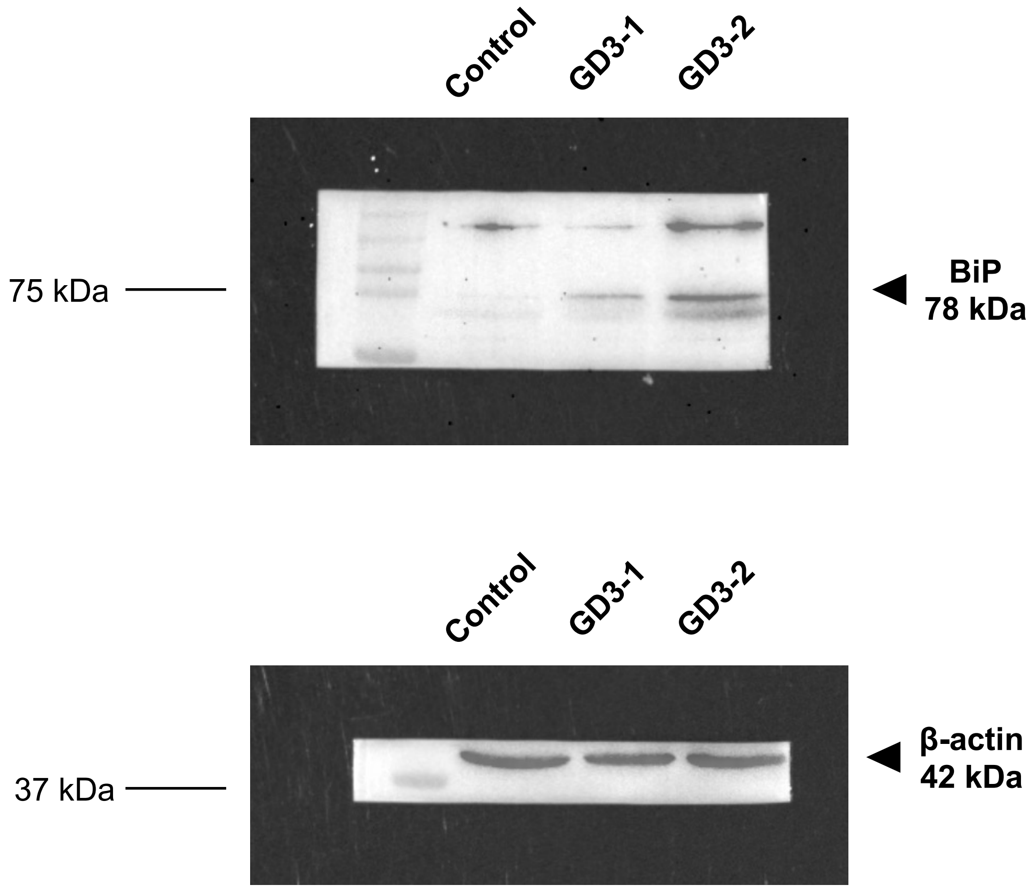
Control
GD3-1
GD3-2
75 kDa
BiP
78 kDa
Control
GD3-1
GD3-2
37 kDa
β-actin
42 kDa

BiP-4

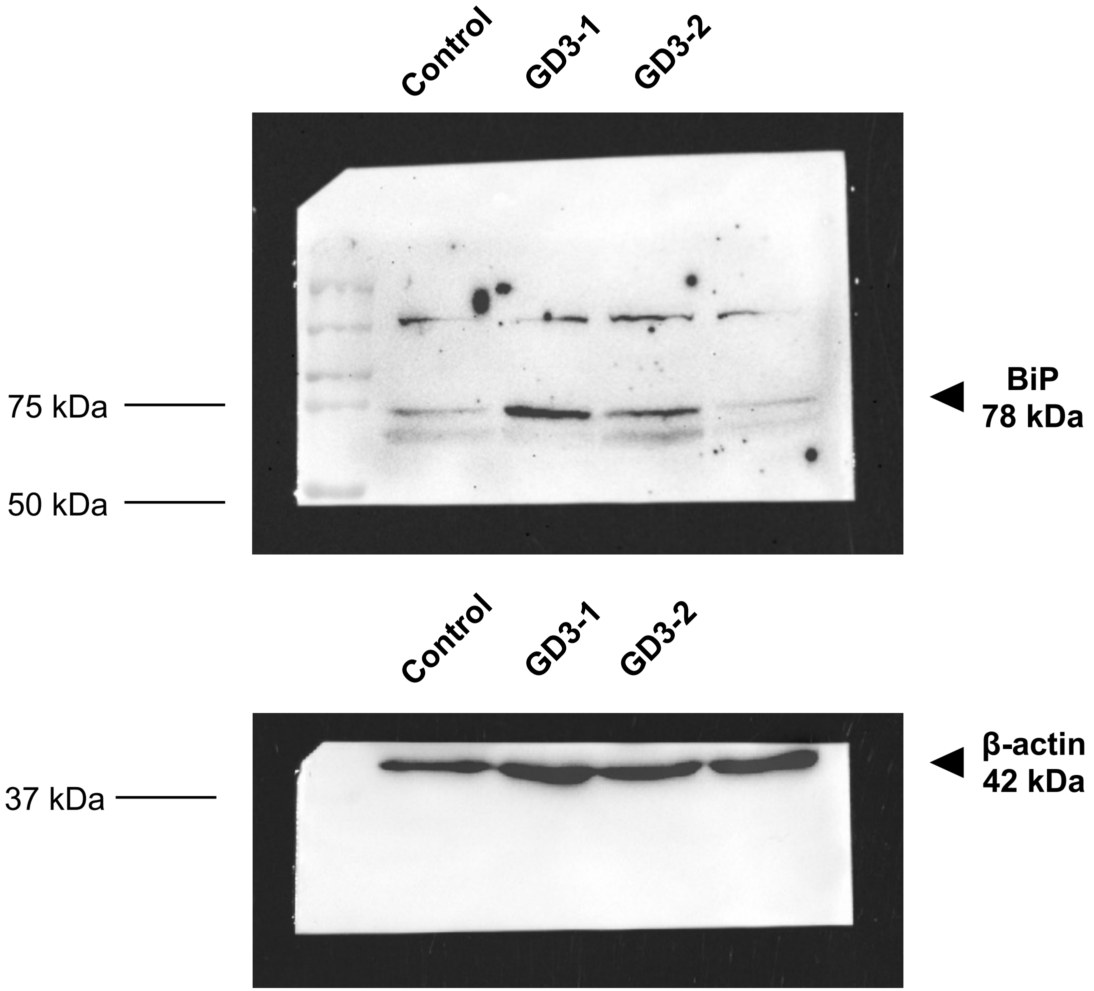

Control
GD3-1
GD3-2
75 kDa
50 kDa
BiP
78 kDa
Control
GD3-1
GD3-2
37 kDa
β-actin
42 kDa

BiP-5

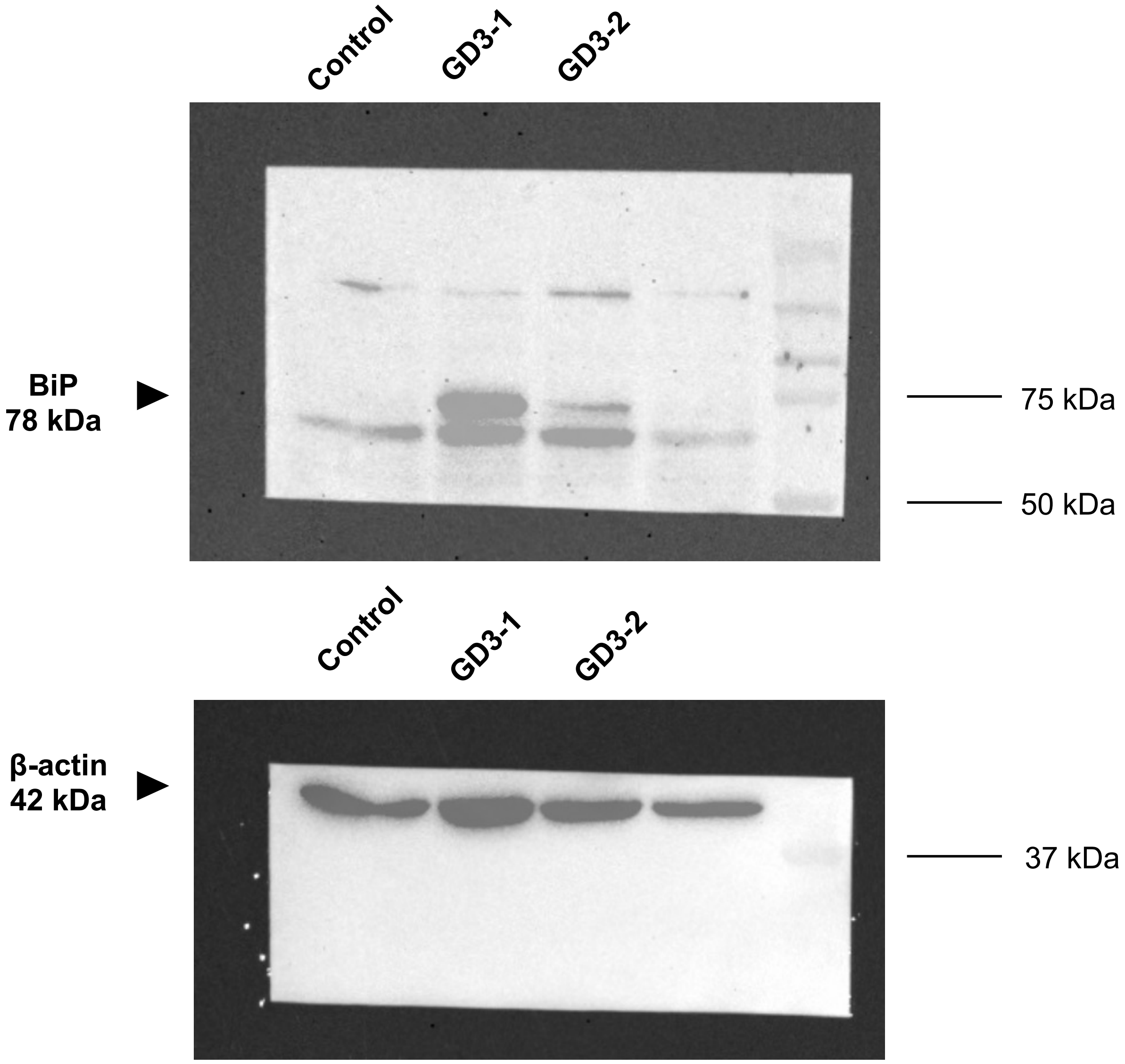
Control
GD3-1
GD3-2
BiP
78 kDa
75 kDa
50 kDa
Control
GD3-1
GD3-2
β-actin
42 kDa
37 kDa

BiP-6

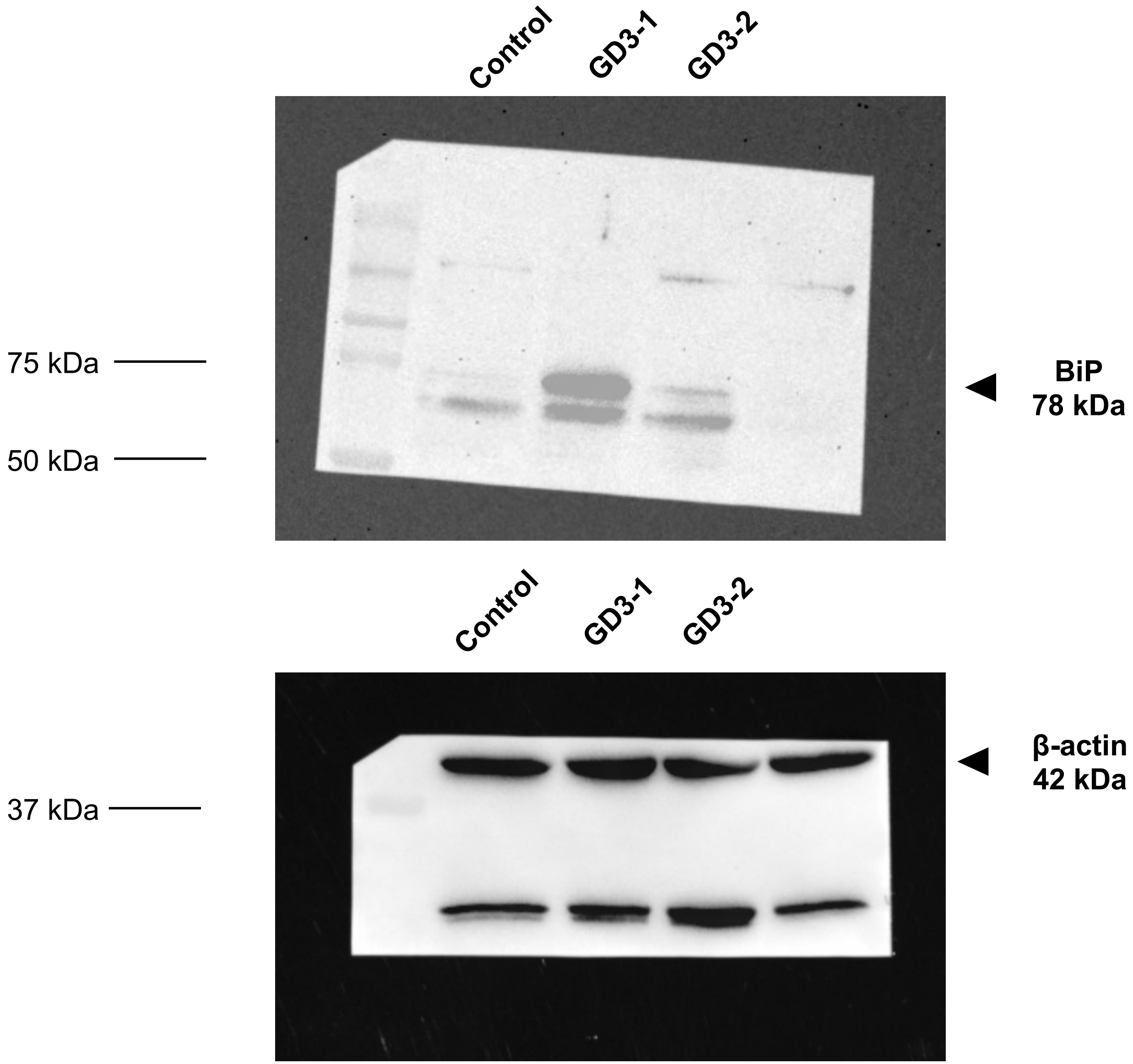

Control
GD3-1
GD3-2
75 kDa
50 kDa
BiP
78 kDa
Control
GD3-1
GD3-2
37 kDa
β-actin
42 kDa

BiP-7

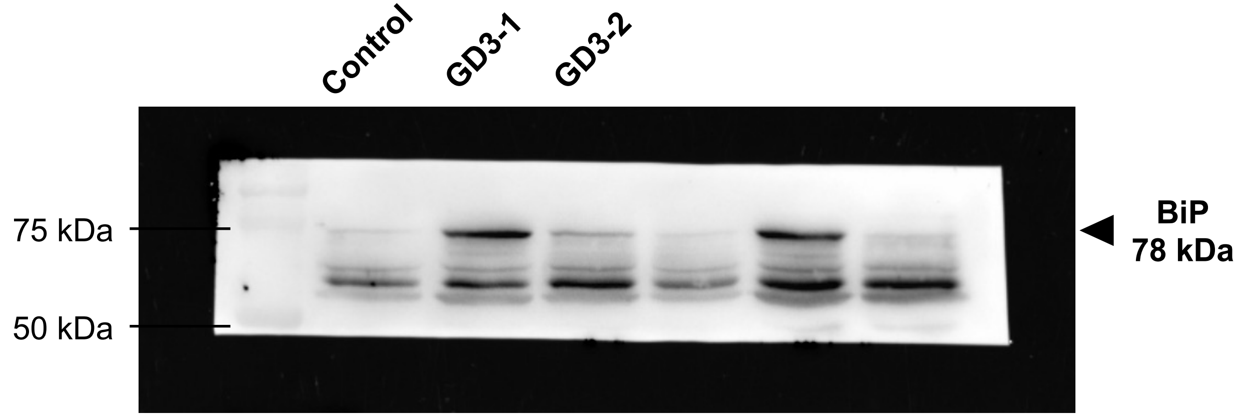
Control
GD3-1
GD3-2
75 kDa
50 kDa
BiP
78 kDa

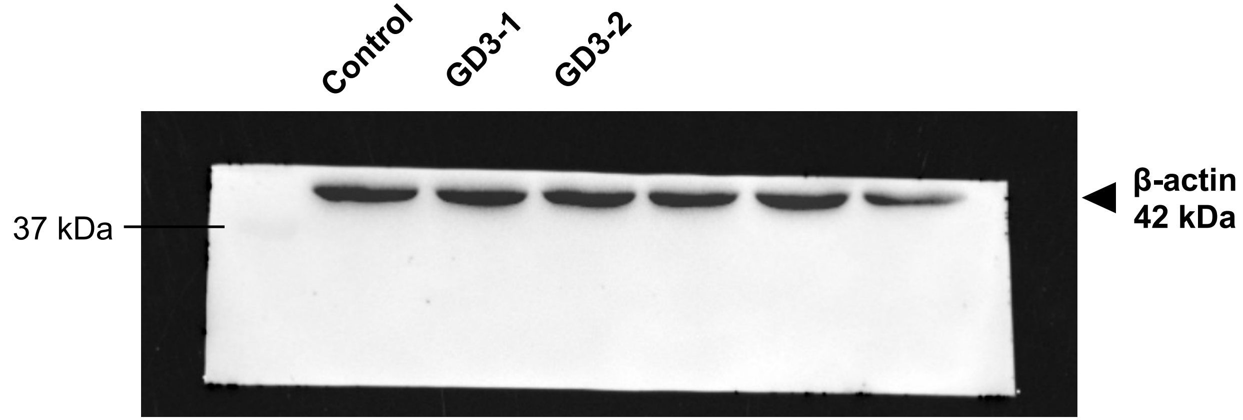
Control
GD3-1
GD3-2
37 kDa
β-actin
42 kDa

CHOP-1

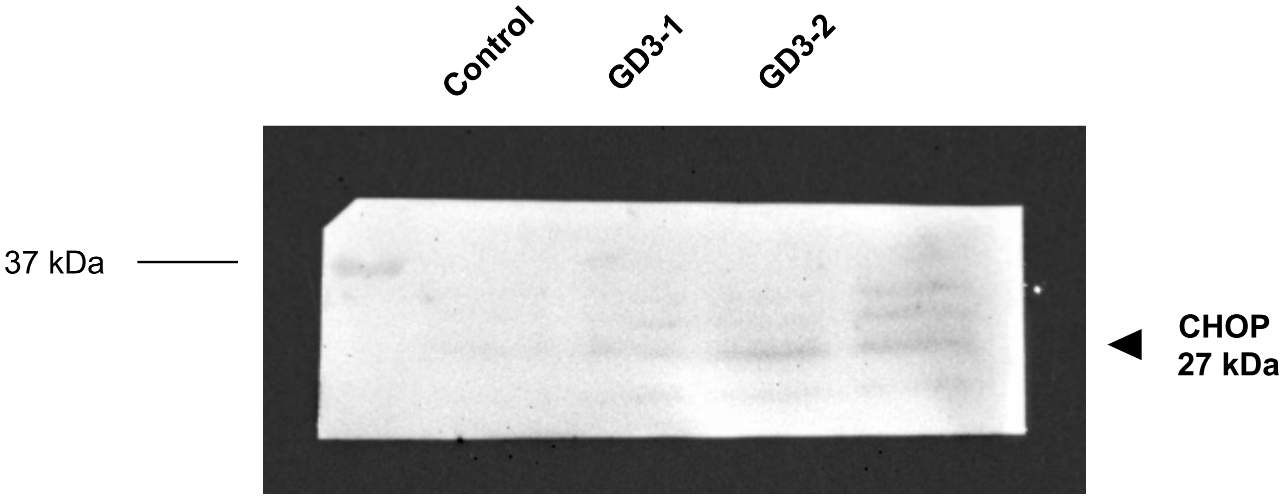
Control
GD3-1
GD3-2
37 kDa
CHOP
27 kDa

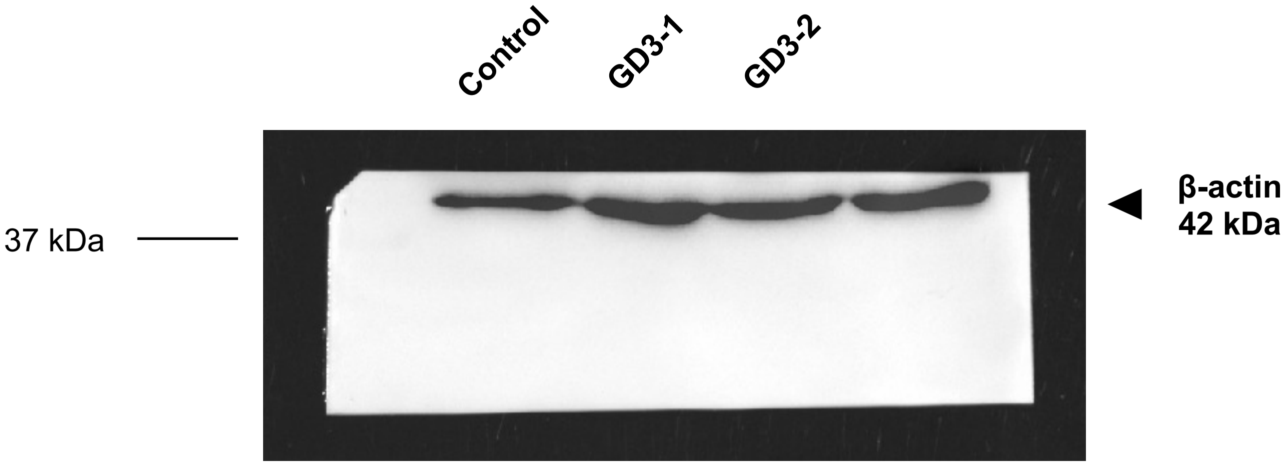
Control
GD3-1
GD3-2
37 kDa
β-actin
42 kDa

CHOP-2

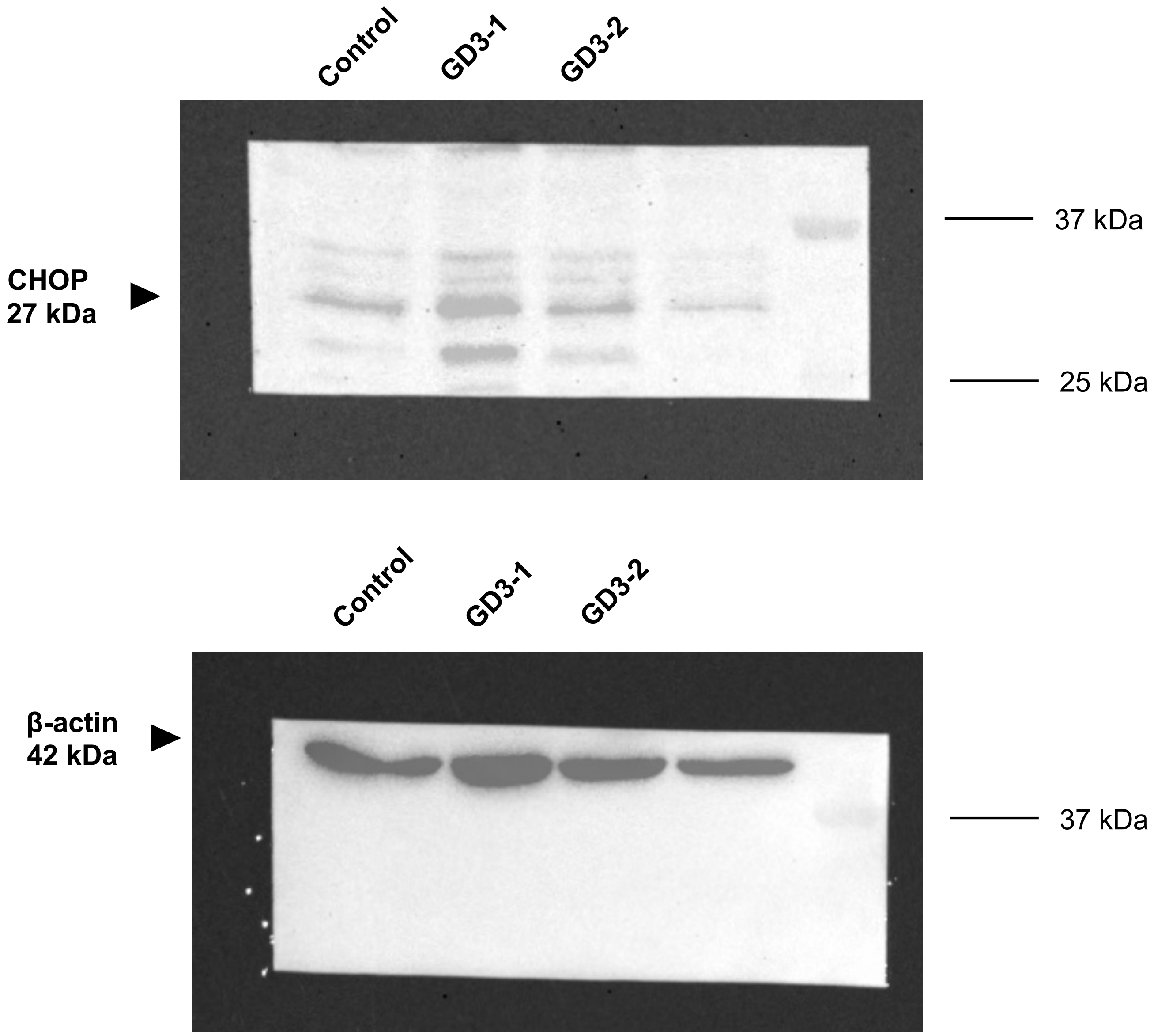

Control
GD3-1
GD3-2
CHOP
27 kDa
37 kDa
25 kDa
Control
GD3-1
GD3-2
β-actin
42 kDa
37 kDa

CHOP-3

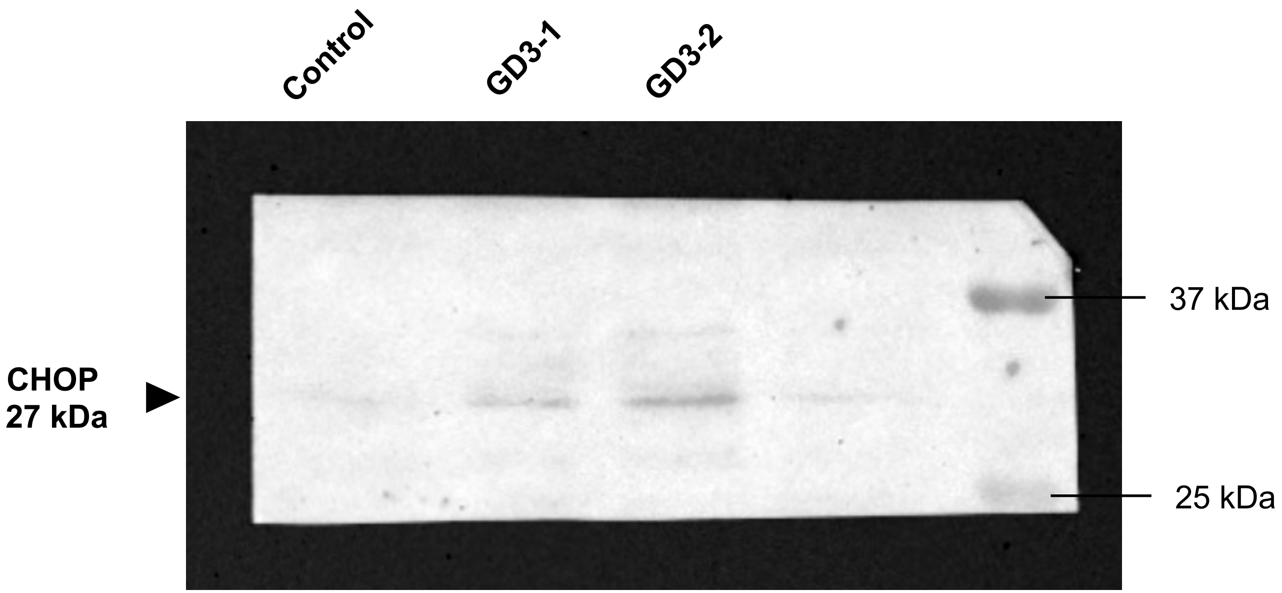
Control
GD3-1
GD3-2
CHOP
27 kDa
37 kDa
25 kDa

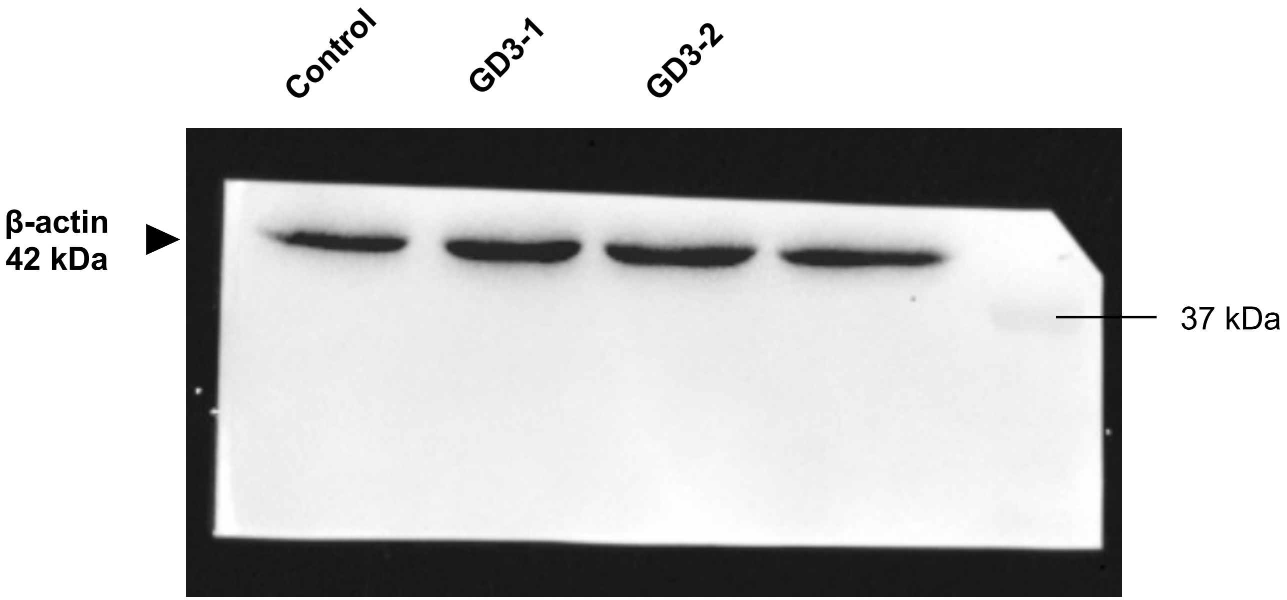
Control
GD3-1
GD3-2
β-actin
42 kDa
37 kDa

CHOP-4

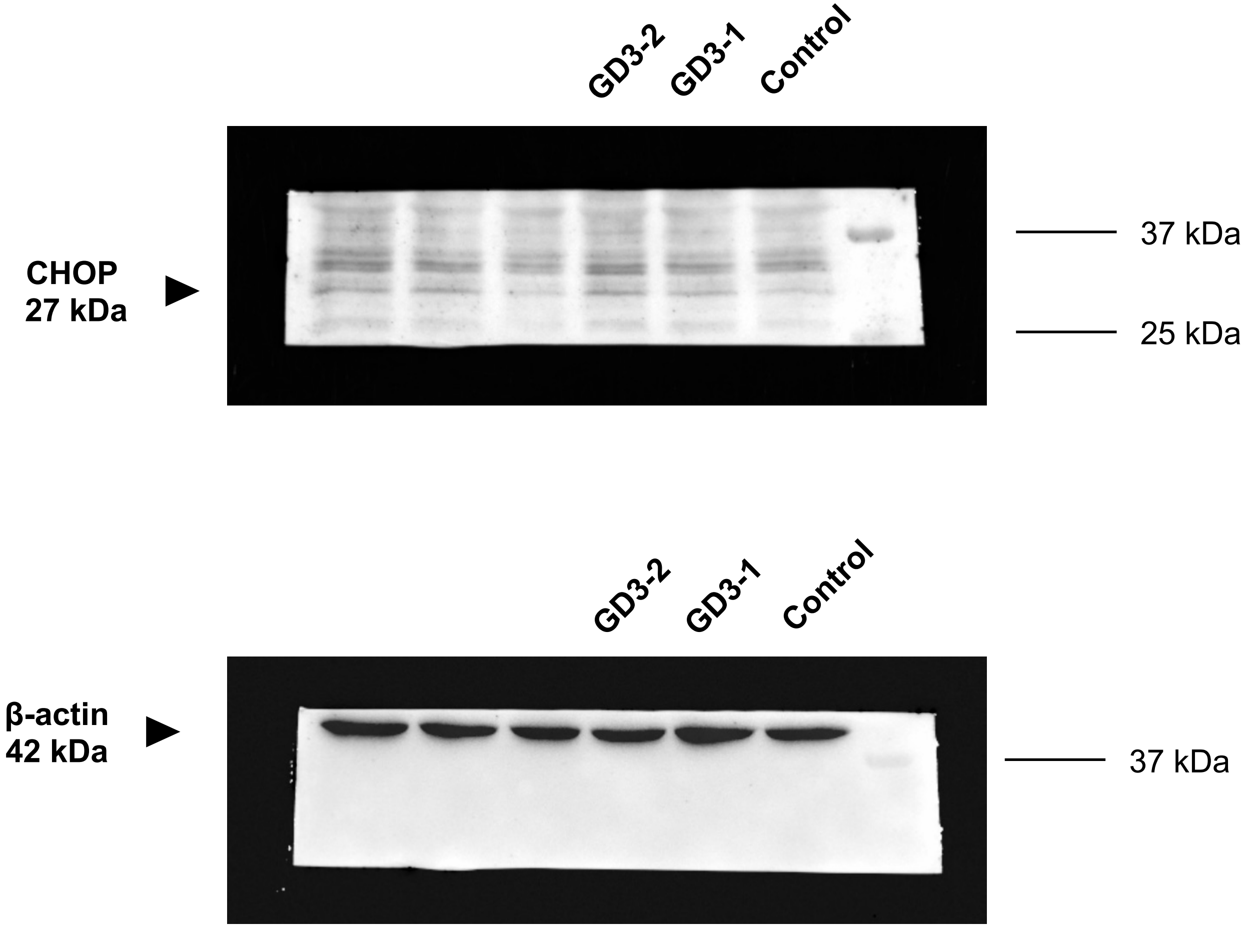
GD3-2
GD3-1
Control
CHOP
27 kDa
37 kDa
25 kDa
GD3-2
GD3-1
Control
β-actin
42 kDa
37 kDa

CHOP-5

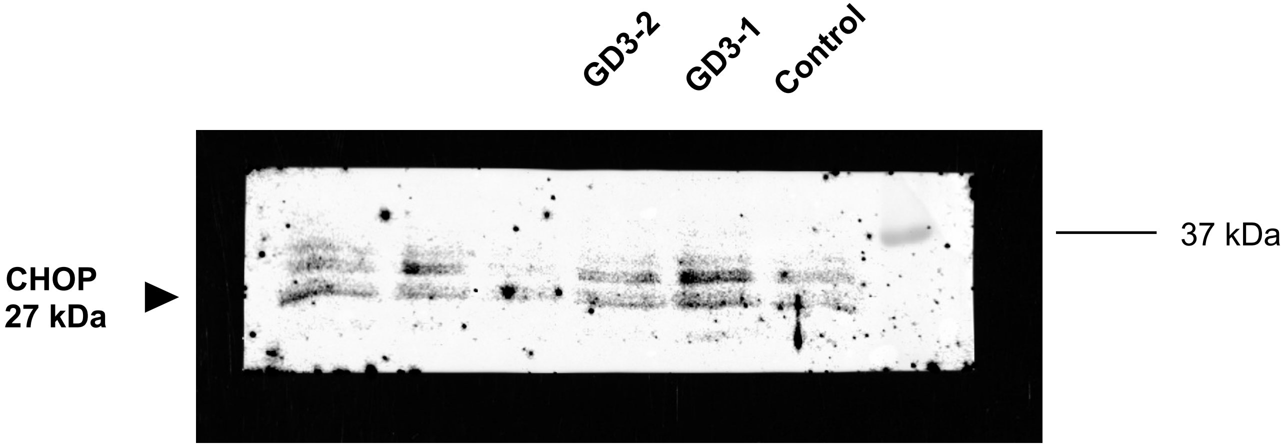
GD3-2
GD3-1
Control
CHOP
27 kDa
37 kDa

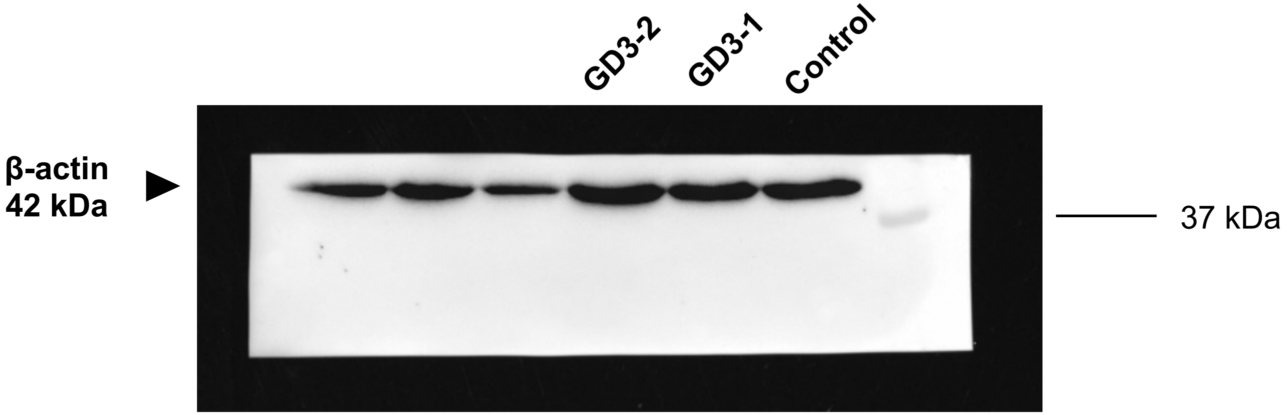
GD3-2
GD3-1
Control
β-actin
42 kDa
37 kDa

BAX-1

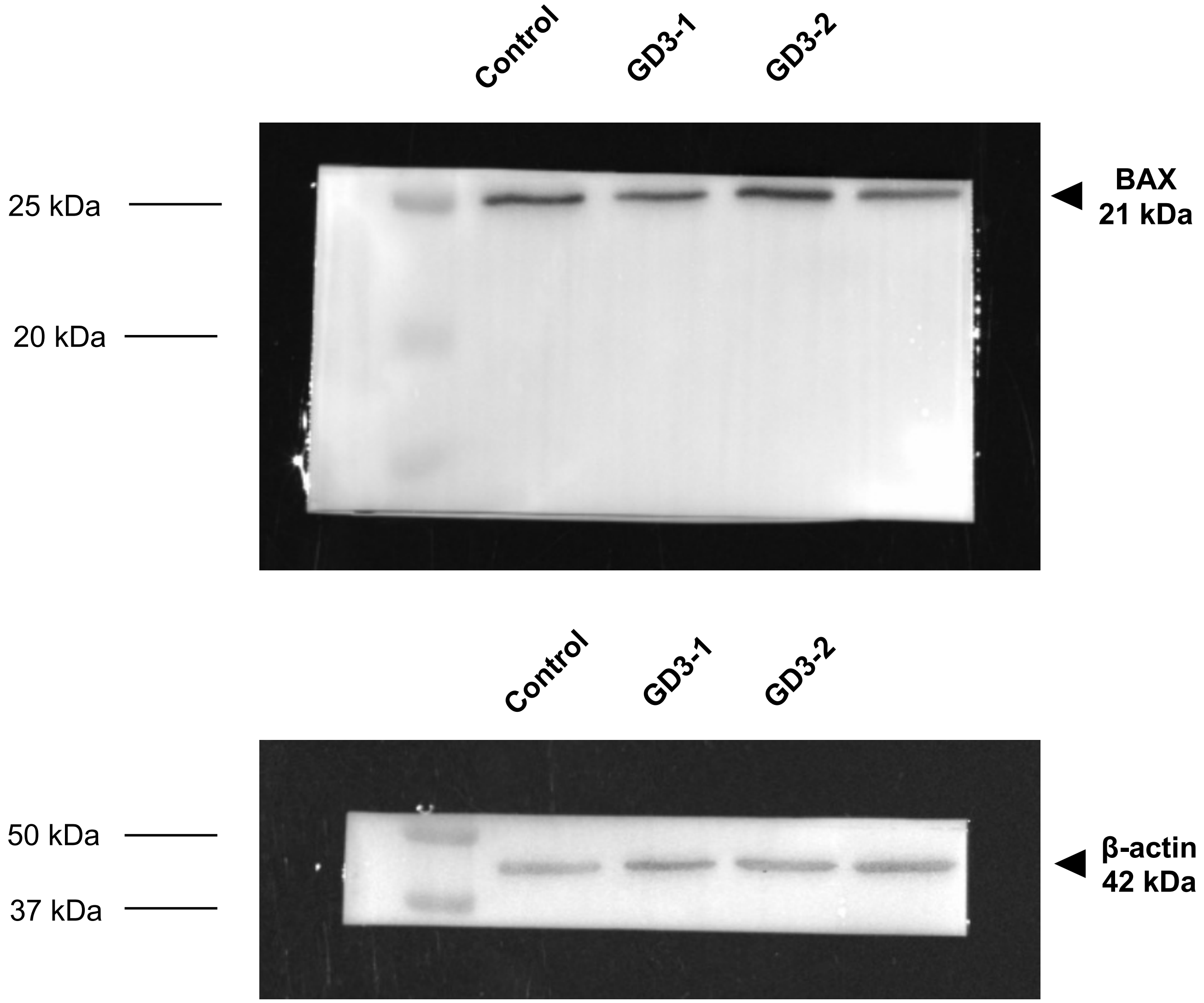
Control
GD3-1
GD3-2
25 kDa
20 kDa
BAX
21 kDa
Control
GD3-1
GD3-2
50 kDa
37 kDa
β-actin
42 kDa

BAX-2

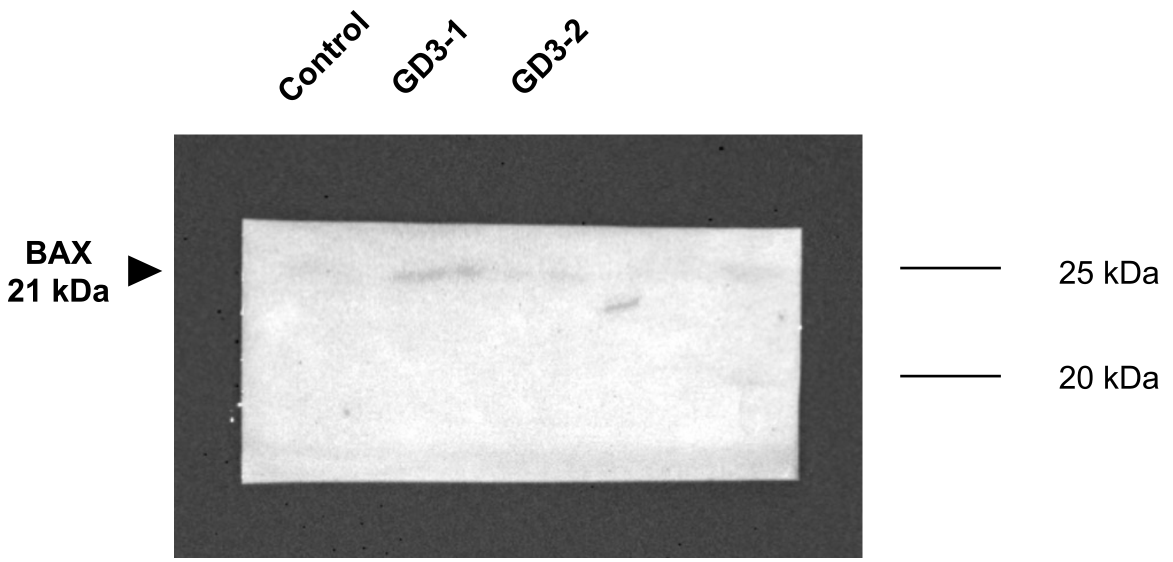
Control
GD3-1
GD3-2
BAX
21 kDa
25 kDa
20 kDa

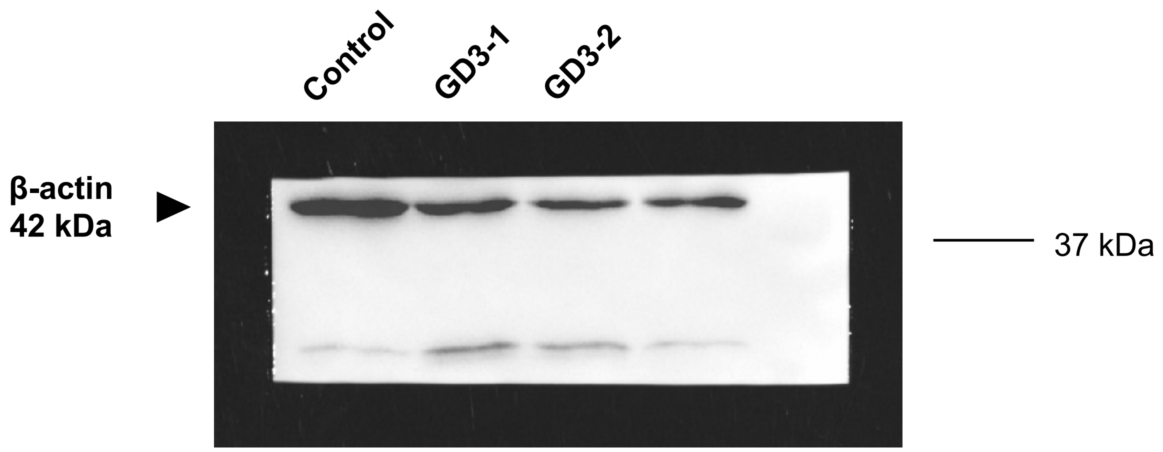
Control
GD3-1
GD3-2
β-actin
42 kDa
37 kDa

BAX-3

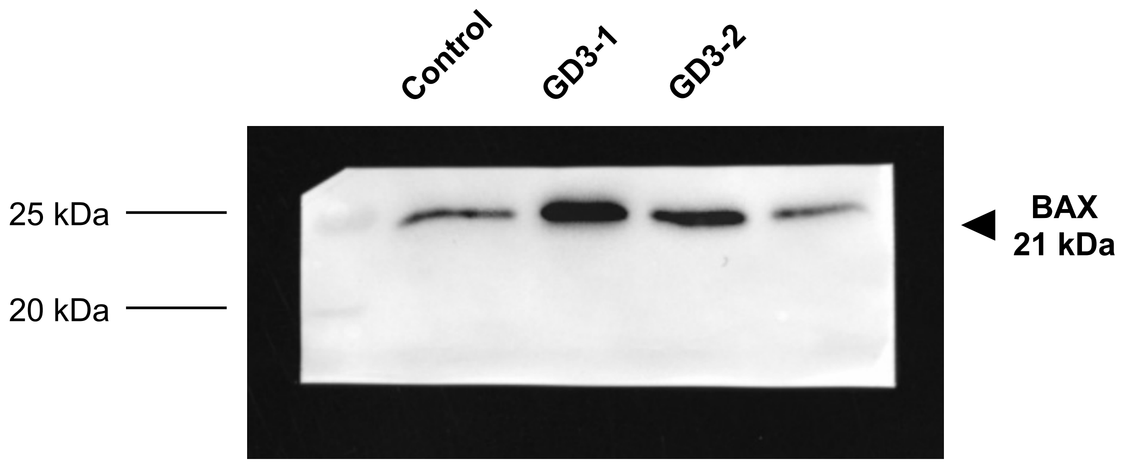
Control
GD3-1
GD3-2
25 kDa
20 kDa
BAX
21 kDa

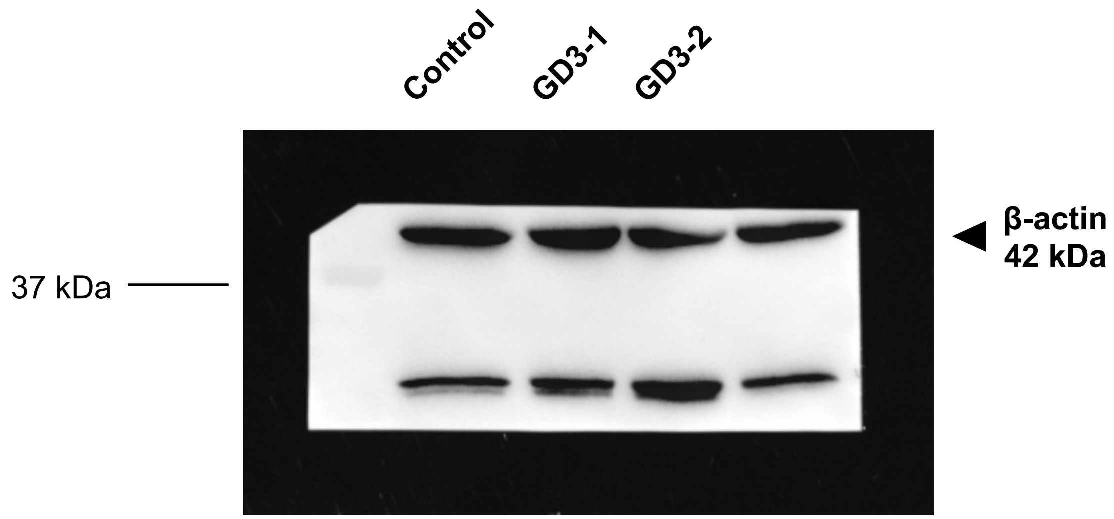
Control
GD3-1
GD3-2
37 kDa
β-actin
42 kDa

BAX-4

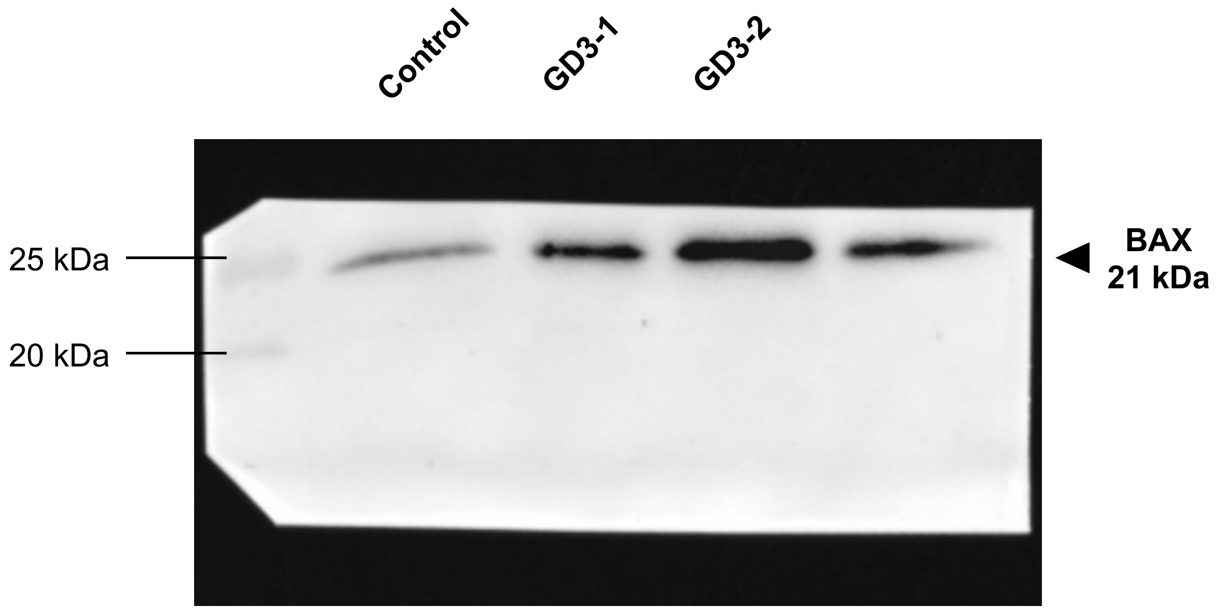
Control
GD3-1
GD3-2
25 kDa
20 kDa
BAX
21 kDa

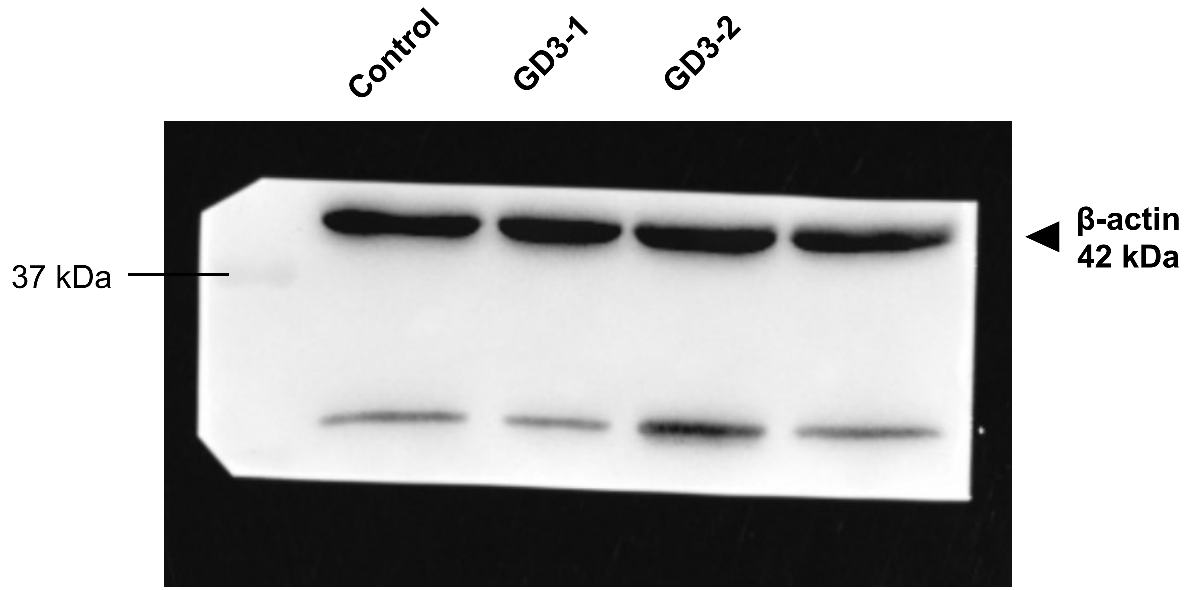
Control
GD3-1
GD3-2
37 kDa
β-actin
42 kDa

BAX-5

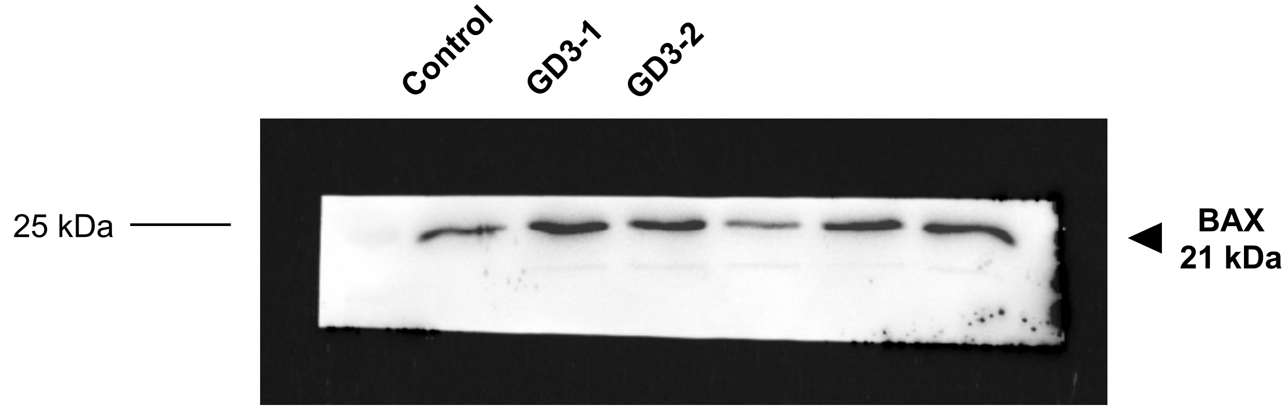
Control
GD3-1
GD3-2
25 kDa
BAX
21 kDa

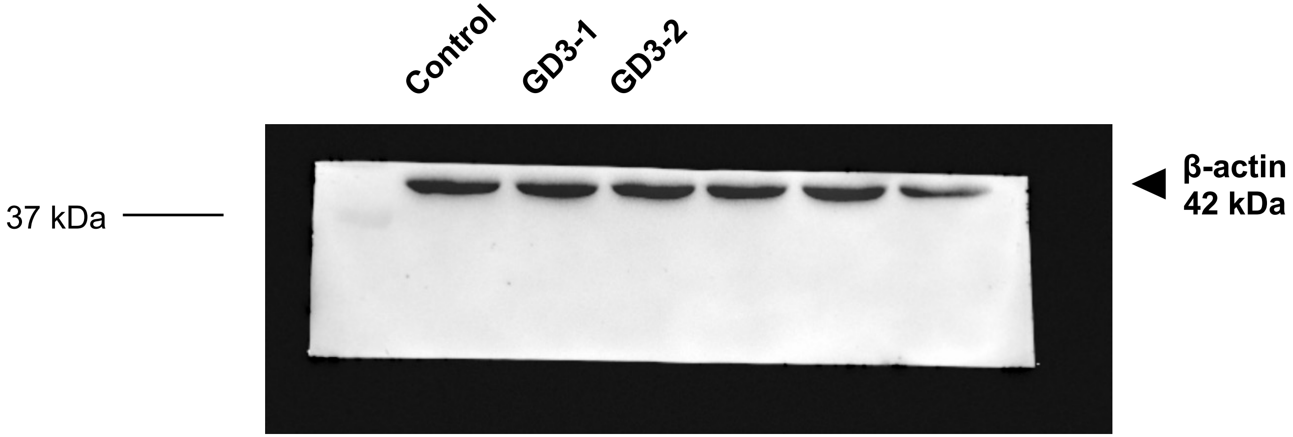
Control
GD3-1
GD3-2
37 kDa
β-actin
42 kDa

BAX-6

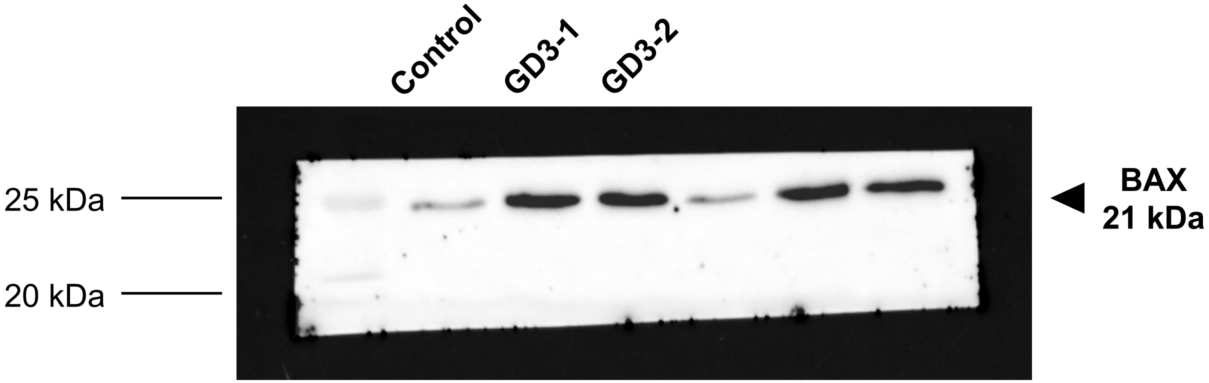
Control
GD3-1
GD3-2
25 kDa
20 kDa
BAX
21 kDa

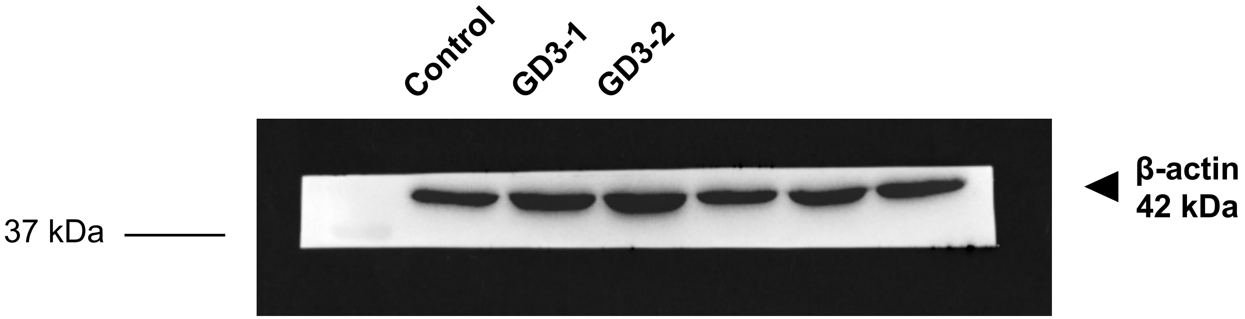
Control
GD3-1
GD3-2
37 kDa
β-actin
42 kDa

BAX-7

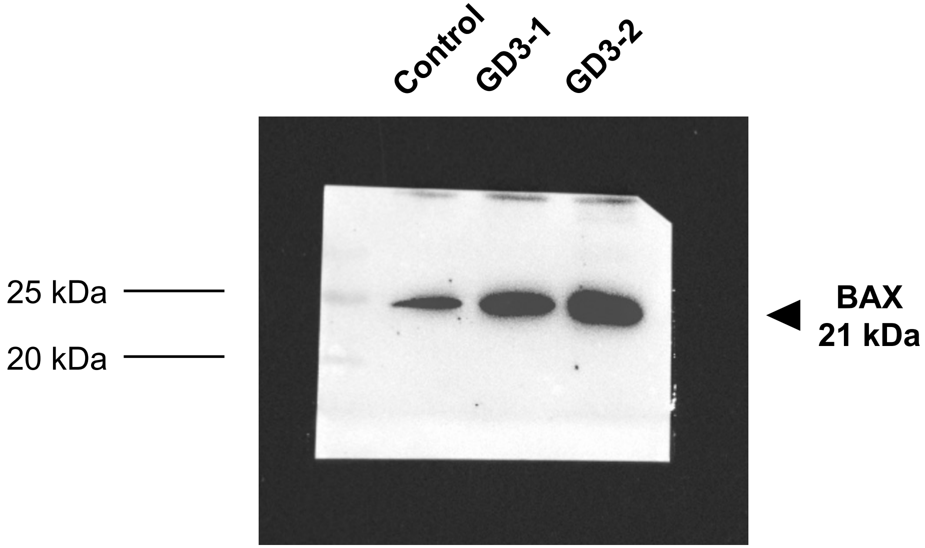
Control
GD3-1
GD3-2
25 kDa
20 kDa
BAX
21 kDa

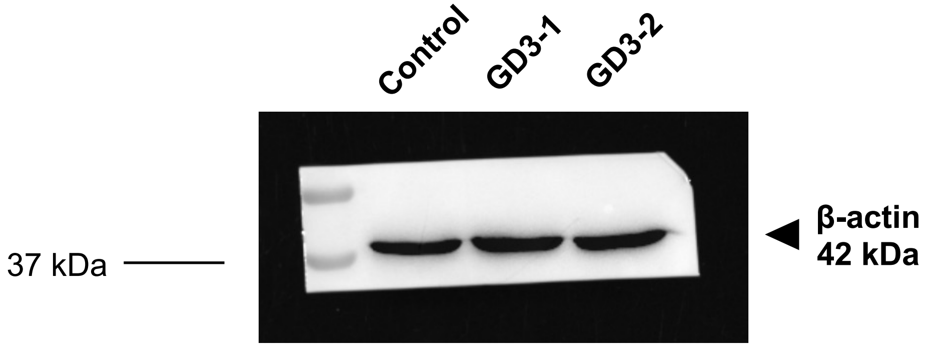
Control
GD3-1
GD3-2
37 kDa
β-actin
42 kDa

BCL-2-1

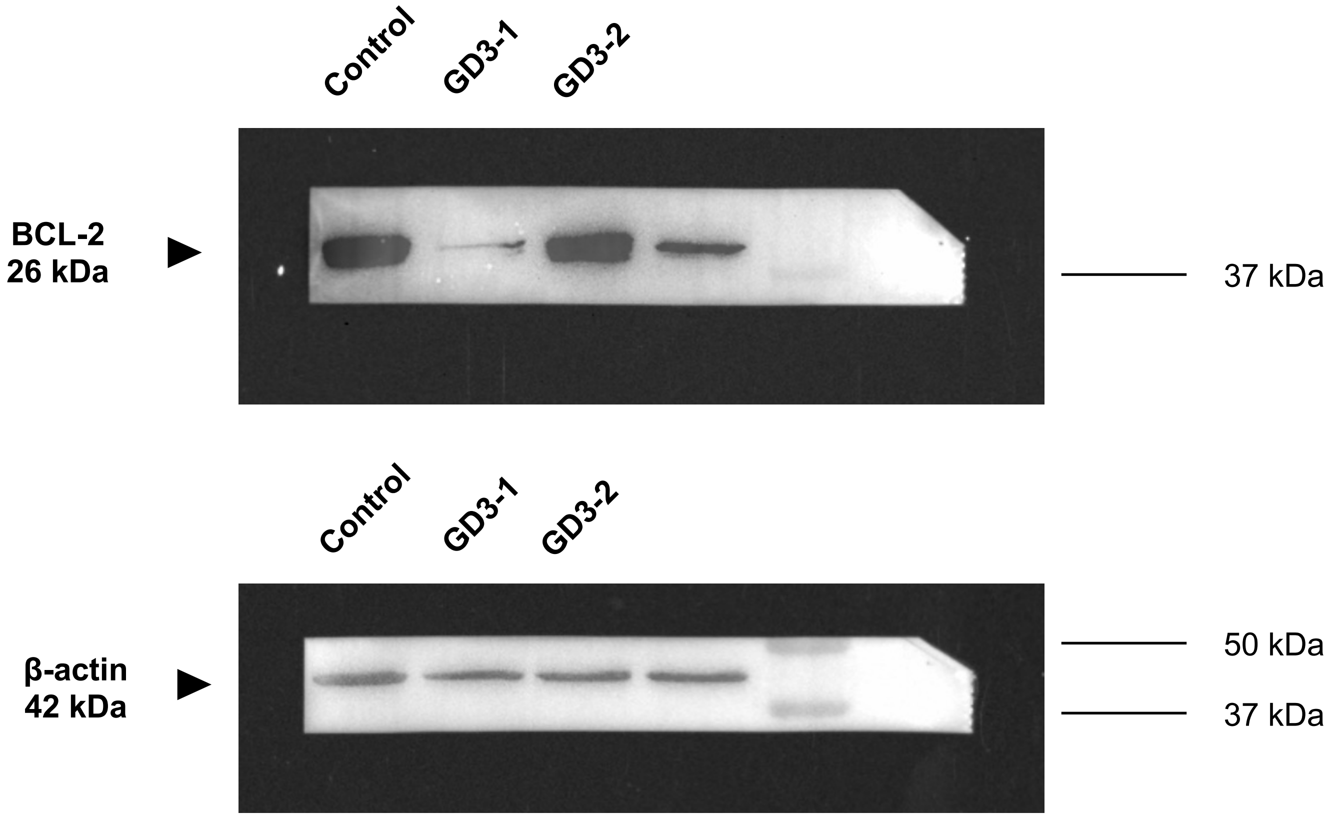
Control
GD3-1
GD3-2
BCL-2
26 kDa
37 kDa
Control
GD3-1
GD3-2
β-actin
42 kDa
50 kDa
37 kDa

BCL-2-2

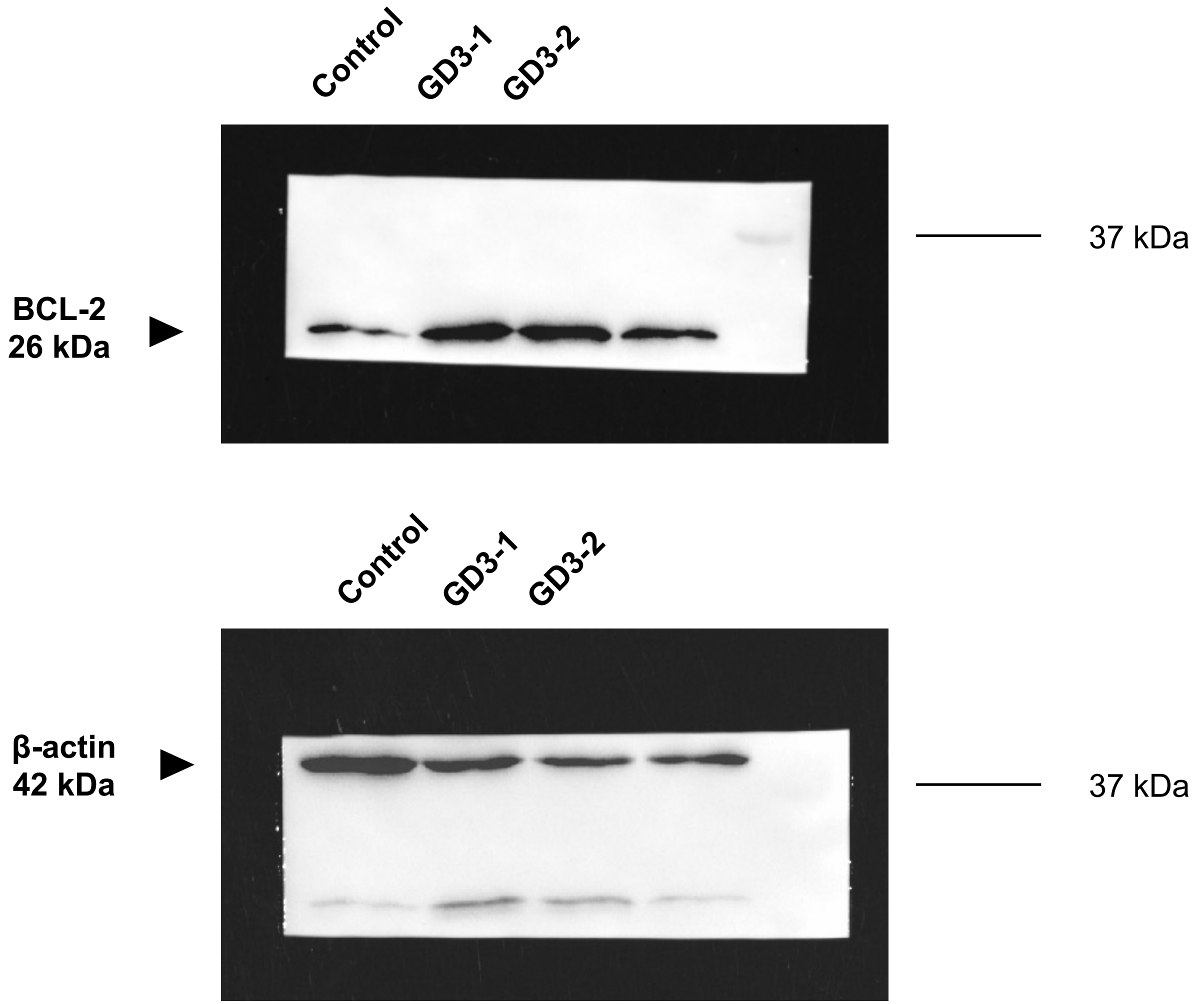

Control
GD3-1
GD3-2
BCL-2
26 kDa
37 kDa
Control
GD3-1
GD3-2
β-actin
42 kDa
37 kDa

BCL-2-3

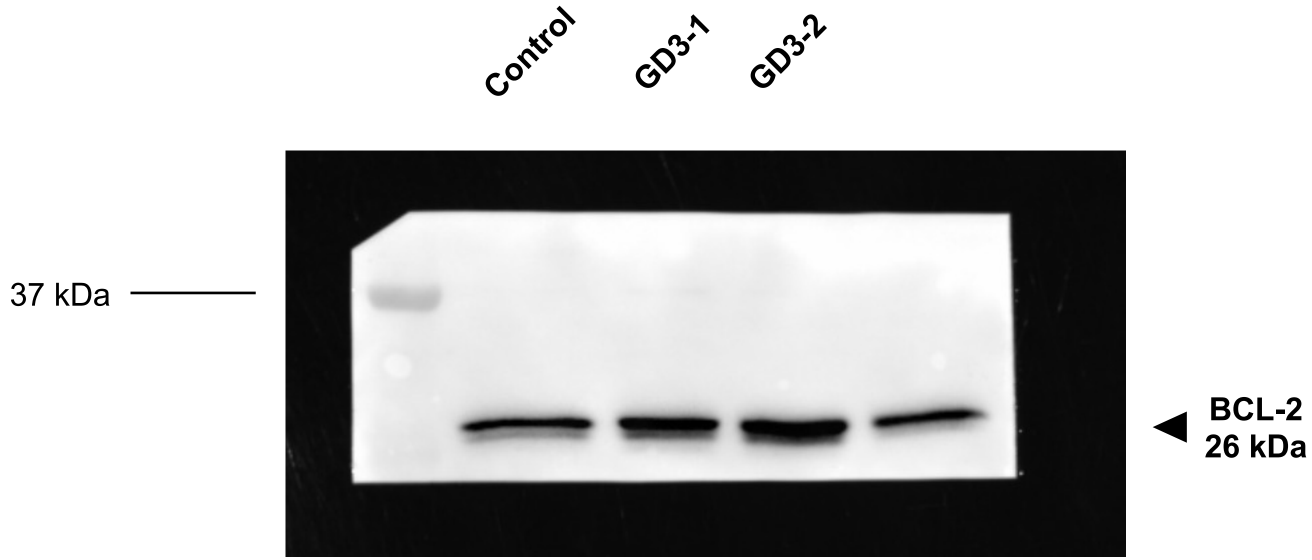
Control
GD3-1
GD3-2
37 kDa
BCL-2
26 kDa

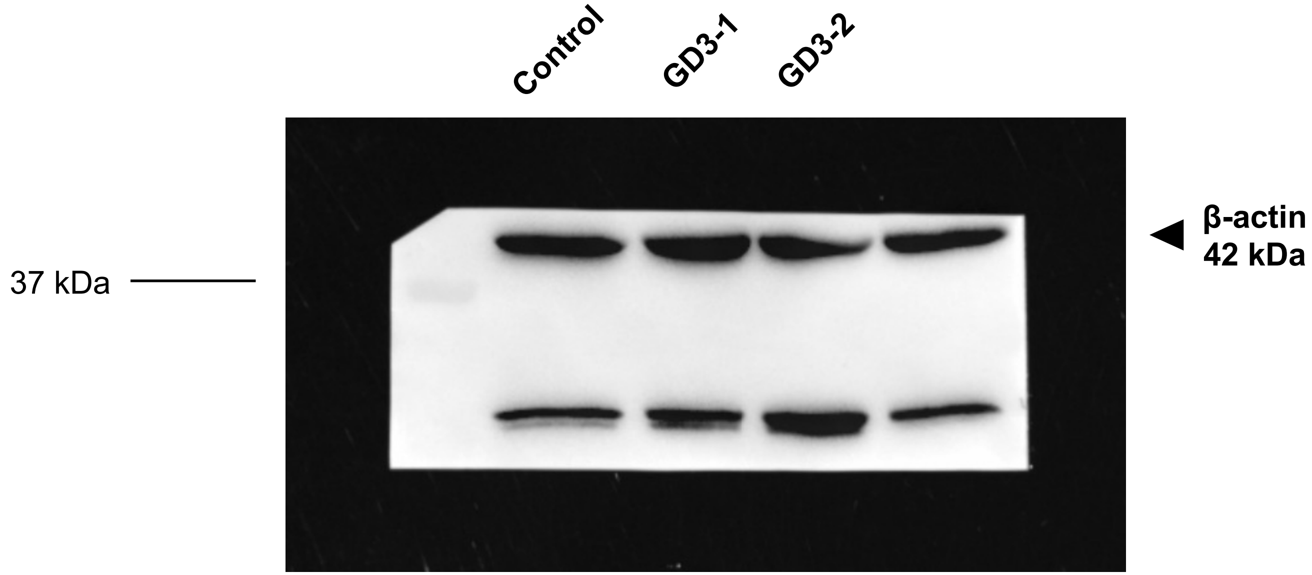
Control
GD3-1
GD3-2
37 kDa
β-actin
42 kDa

BCL-2-4

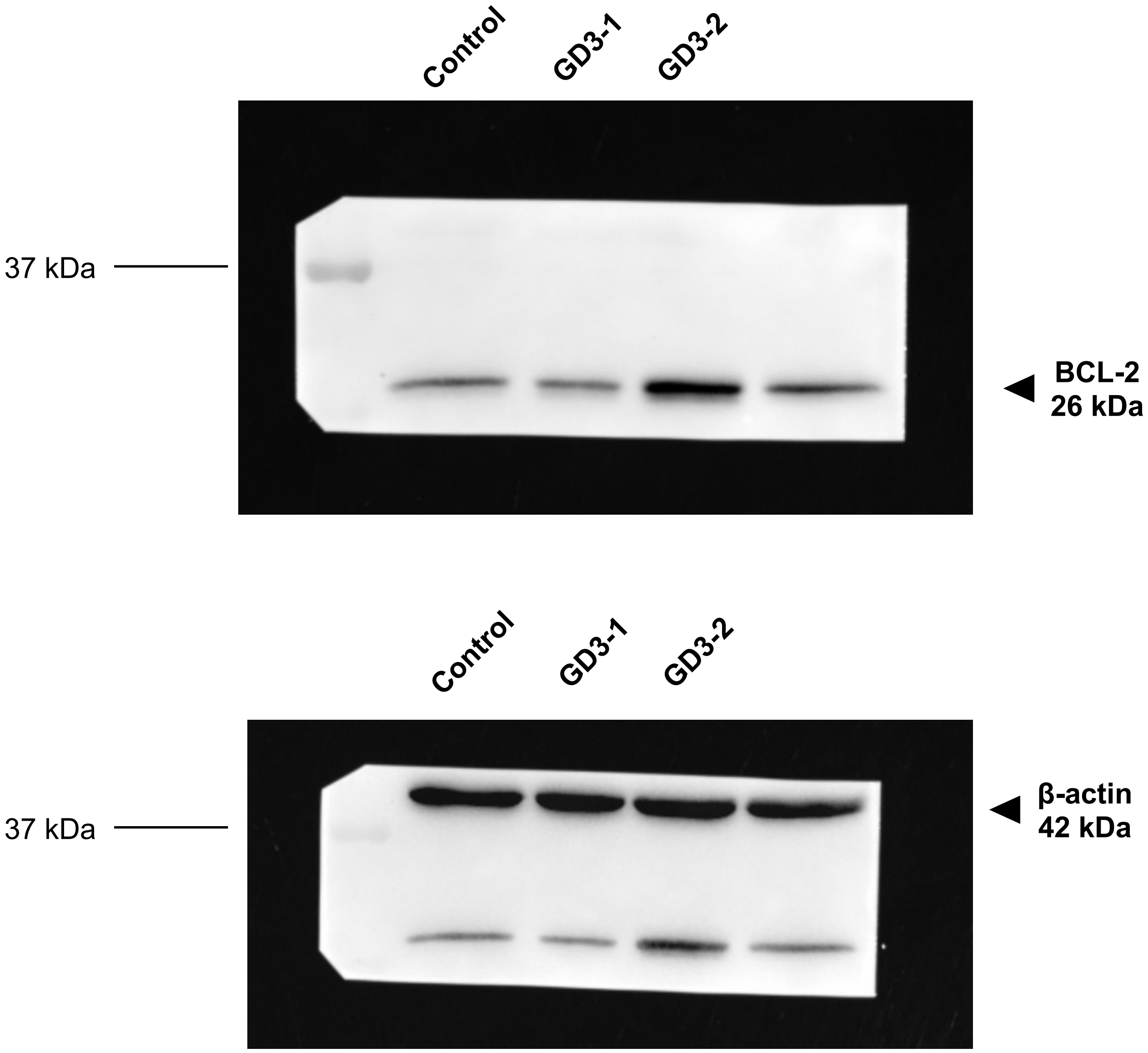
Control
GD3-1
GD3-2
37 kDa
BCL-2
26 kDa
Control
GD3-1
GD3-2
37 kDa
β-actin
42 kDa

BCL-2-5

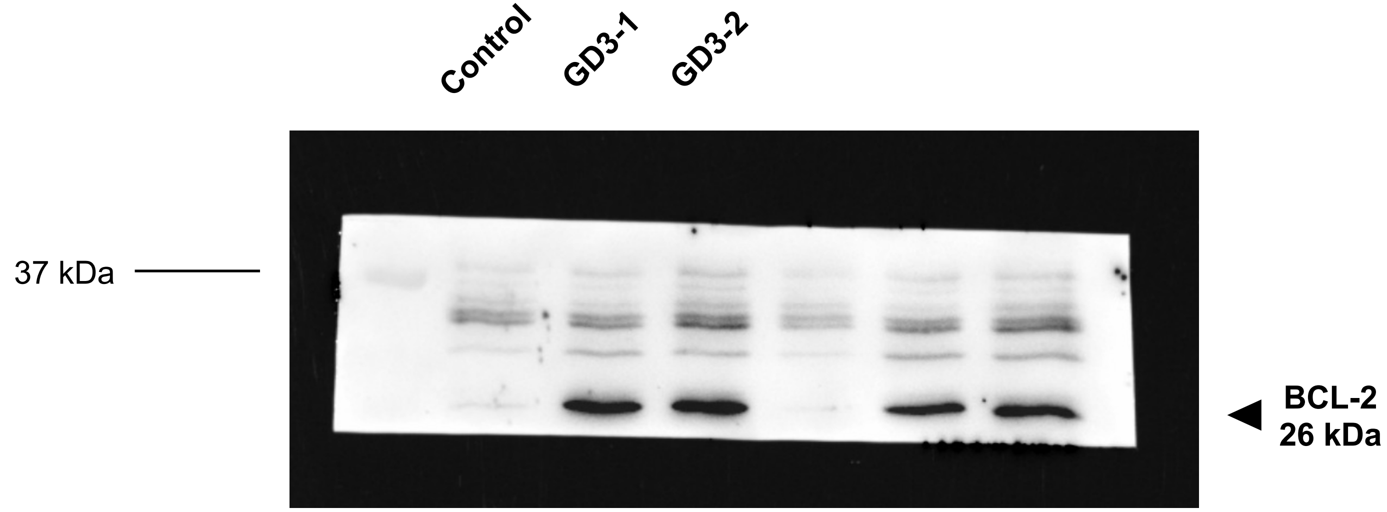
Control
GD3-1
GD3-2
37 kDa
BCL-2
26 kDa

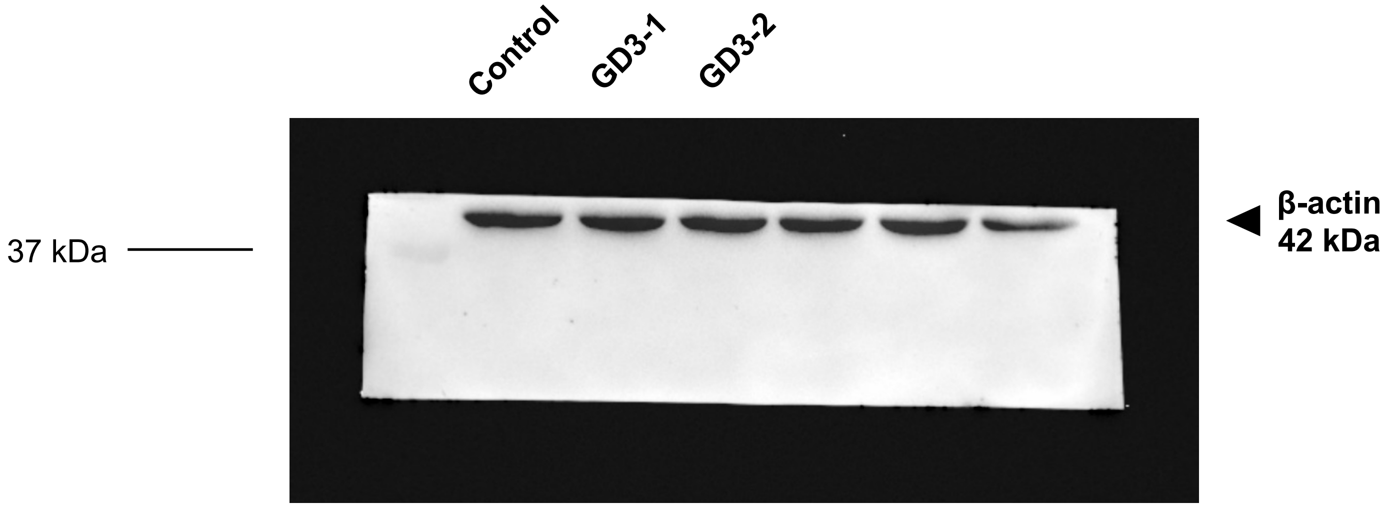
Control
GD3-1
GD3-2
37 kDa
β-actin
42 kDa

BCL-2-6

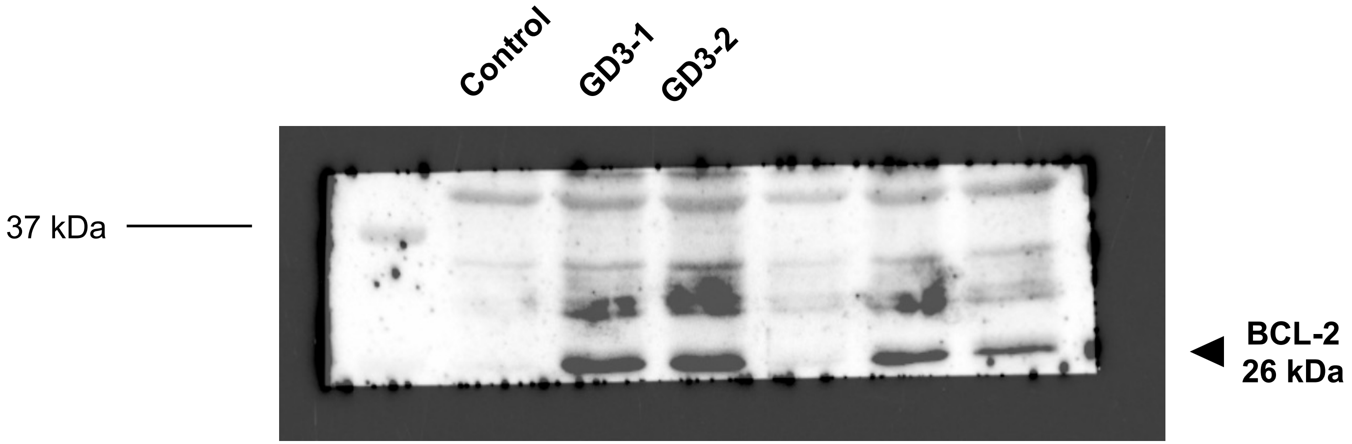
Control
GD3-1
GD3-2
37 kDa
BCL-2
26 kDa

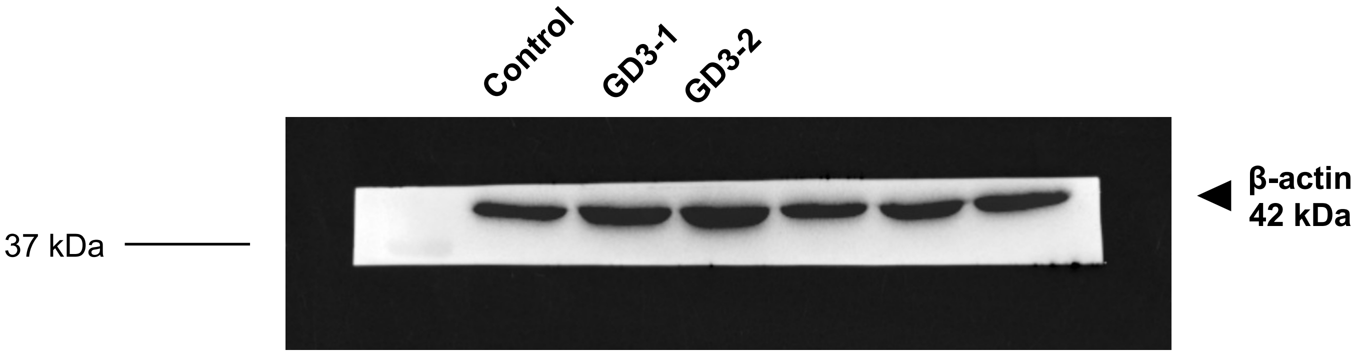
Control
GD3-1
GD3-2
37 kDa
β-actin
42 kDa

BCL-2-7

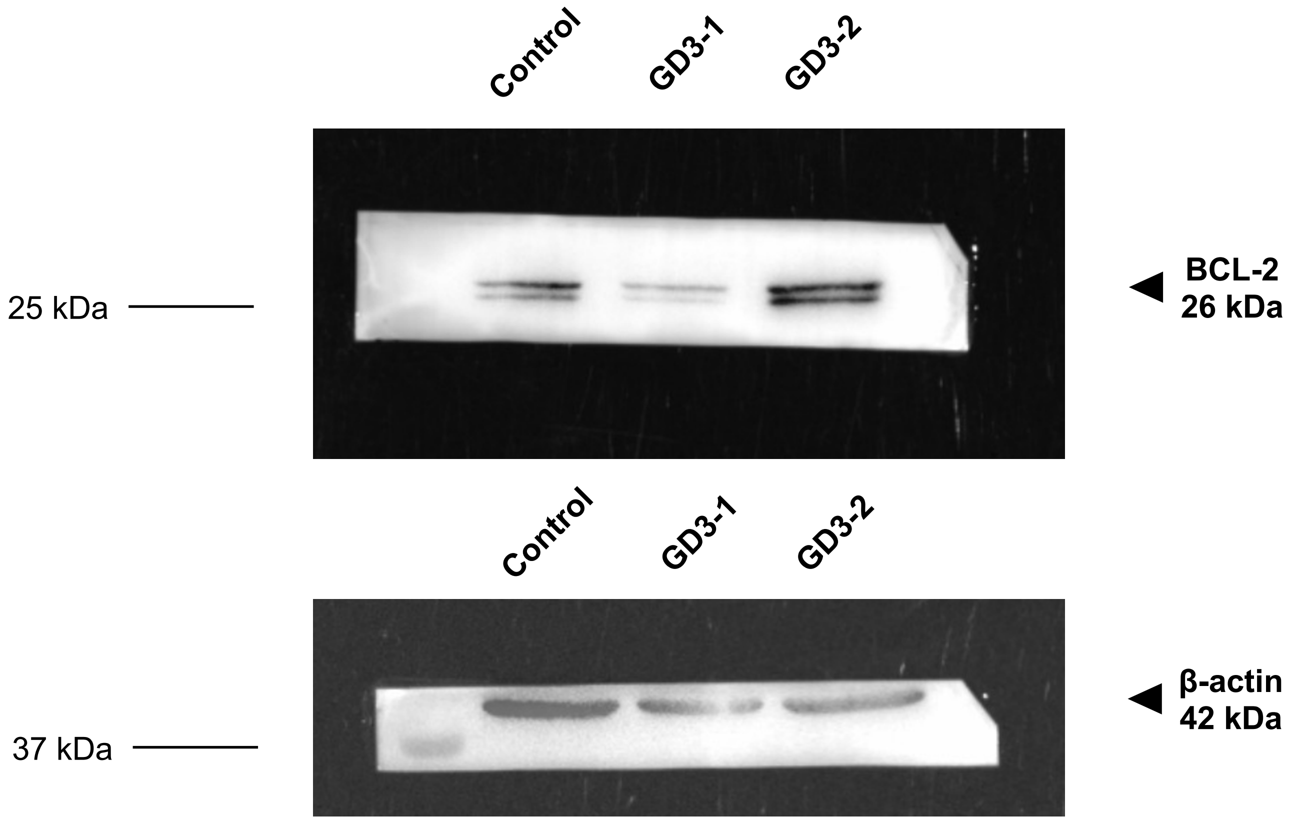

Control
GD3-1
GD3-2
25 kDa
BCL-2
26 kDa
Control
GD3-1
GD3-2
37 kDa
β-actin
42 kDa
